# Population genetic structure and evolutionary history of *Psammochloa villosa* (Trin.) Bor (Poaceae) revealed by AFLP marker

**DOI:** 10.1002/ece3.7831

**Published:** 2021-07-13

**Authors:** Ting Lv, AJ Harris, Yuping Liu, Tao Liu, Ruifang Liang, Zilan Ma, Xu Su

**Affiliations:** ^1^ School of Geosciences Qinghai Normal University Xining China; ^2^ Academy of Plateau Science and Sustainability Xining China; ^3^ Key Laboratory of Plant Resources Conservation and Sustainable Utilization South China Botanical Garden Chinese Academy of Sciences Guangzhou China; ^4^ Key Laboratory of Medicinal Animal and Plant Resources of the Qinghai‐Tibet Plateau in Qinghai Province School of Life Science Qinghai Normal University Xining China; ^5^ Key Laboratory of Education Ministry of Earth Surface Processes and Ecological Conservation of the Qinghai‐Tibet Plateau Qinghai Normal University Xining China

**Keywords:** desert grasslands, ecological niche modeling, Inner Mongolian Plateau, population genetics, SAMOVA

## Abstract

*Psammochloa* *villosa* is an ecologically important desert grass that occurs in the Inner Mongolian Plateau where it is frequently the dominant species and is involved in sand stabilization and wind breaking. We sought to generate a preliminary demographic framework for *P. villosa* to support the future studies of this species, its conservation, and sustainable utilization. To accomplish this, we characterized the genetic diversity and structure of 210 individuals from 43 natural populations of *P. villosa* using amplified fragment length polymorphism (AFLP) markers. We obtained 1,728 well‐defined amplified bands from eight pairs of primers, of which 1,654 bands (95.7%) were polymorphic. Results obtained from the AFLPs suggested effective alleles among populations of 1.32, a Nei's standard genetic distance value of 0.206, a Shannon index of 0.332, a coefficient of gene differentiation (*G*_ST_) of 0.469, and a gene flow parameter (*N*m) of 0.576. All these values indicate that there is abundant genetic diversity in *P. villosa*, but limited gene flow. An analysis of molecular variance (AMOVA) showed that genetic variation mainly exists within populations (64.2%), and we found that the most genetically similar populations were often not geographically adjacent. Thus, this suggests that the mechanisms of gene flow are surprisingly complex in this species and may occur over long distances. In addition, we predicted the distribution dynamics of *P. villosa* based on the spatial distribution modeling and found that its range has contracted continuously since the last interglacial period. We speculate that dry, cold climates have been critical in determining the geographic distribution of *P. villosa* during the Quaternary period. Our study provides new insights into the population genetics and evolutionary history of *P. villosa* in the Inner Mongolian Plateau and provides a resource that can be used to design in situ conservation actions and prioritize sustainable utilization.

## Graphical abstract

1. There is abundant genetic diversity in *Psammochloa villosa*, but limited gene flow. 2. Within *P. villosa*, there is limited but noteworthy geographic structuring of populations, of which 64.2% show significant genetic variation. 3. The species experienced continuous range contraction since the last interglacial period, and the dry and cold climates were critical in determining the distribution of *P. villosa* during the Quaternary period.

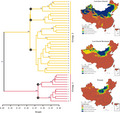

## 1 INTRODUCTION

The Quaternary period, comprising the Holocene and Pleistocene Epochs, spanned the last ~2.6 million years (Myr) and has been characterized by distinct climatic oscillations, especially alternating glacial and interglacial cycles in the Northern Hemisphere (Elias, 2013). The glacial cycles covaried with, and probably profoundly affected, other aspects of the climate, including the intensity of the Asian monsoon, even in unglaciated regions (An et al., 2001; Liu et al., 2018). Climate fluctuations during the Quaternary glaciations led to dramatic changes in the geographic distribution, genetic structure, and population demography of plant species (Jia et al., 2012; Liu et al., 2018).

Quaternary climate change is known to have strongly affected the distributions of plants in Northern China (Liu et al., 2012). Northern China was dominated by deserts, which likely developed during the Quaternary based on geological interpretations of deposits of silt‐sized sediment (i.e., loess) (Sun et al., 2018). The process of desert formation in Northern China was once understood to be a result of sustained orogenesis of the Qinghai‐Tibetan Plateau and surrounding areas during the Quaternary (Meng & Zhang, 2011; Wu, 2002). However, new data showed that the plateau reached its height earlier than originally thought in the Miocene (Hu et al., 2020; Staisch et al., 2020) and suggested that the deserts might have originated during glacial periods, when global ice volume was high and, thus, liquid water was more limited (Sun et al., 2018). The same climatic processes that gave rise to the deserts also appear to have profoundly shaped regional plant diversity and yielded highly complex demographic histories of native species (Ge et al., 2005). In particular, many plants within the deserts of Northern China underwent an adaptive and demographic change in response to cold, arid conditions, such that the intermittent glacial periods may be the primary mechanism explaining modern distributions (e.g., El‐Tayeh et al., 2020; Su & Zhang, 2013; Xu & Zhang, 2015). For example, Su and Zhang (2013) proposed that the onset of aridity during Quaternary glacial periods was a primary driver of population processes and structures in *Nitraria sphaerocarpa* Maxim. (Nitrariaceae), and Xu and Zhang (2015) revealed that periods of cold, arid conditions during the Pleistocene glaciations resulted in genetic differentiation and demographic structuring in *Atraphaxis frutescens* (L.) K. Koch (Polygonaceae).

Nevertheless, these prior studies on population histories of desert plants of Northern China have focused primarily on woody species, and studies on herbs of the region are largely lacking. To our knowledge, the only such study on an herb is on *Delphinium naviculare* W. T. Wang (Ranunculaceae) (Zhang & Zhang, 2012), which is endemic at midelevations within the Tianshan Mountains of Xinjiang Province of China (Wang & Warnock, 2001). Herbaceous plants of the deserts of Northern China merit further study because they comprise a vital component of desert plant communities, and studies focusing on dominant grass species are especially warranted (Meng & Zhang, 2011). Herbaceous plants may have been more sensitive to Quaternary climatic oscillations because they differ markedly from woody species in their responses to cold, often via the death of aboveground biomass as part of either an annual or perennial life cycle. Moreover, dominant species likely achieved their present abundances due to their responses to the glacial cycles.

In modern times, deserts and semideserts, such as in Northern China, are extremely fragile ecosystems, the stability of which impacts global environmental conditions and influences climate change (Su, 2013). Although deserts typically have sparse vegetation, plants are critical for maintaining their integrity. In Northern China, the desert grassland ecosystems in particular are becoming rapidly degraded due to long‐term overgrazing and desertification and, simultaneously, the desert is encroaching on arable land within the region (Deng et al., 2014; Li et al., 2004; Wei et al., 2020). These desert grasslands represent a large area within China and adjacent countries and occur at both low and high elevations. Dominant plant species within the grasslands are often psammophytes, which have special adaptations to resist being buried by sand and to tolerate having periodically exposed roots. At the same time, these plants help to anchor sands in place and prevent wind erosion. Therefore, they are critical for promoting environmental stability within the desert grasslands and preventing desert encroachment (Pan, 2006; Zhou et al., 2011).

In this study, we focused on one psammophyte, *Psammochloa villosa* (Trin.) Bor, which was treated in a monotypic genus of tribe Stipeae in Poaceae. This species, commonly called sand whip, is a perennial rhizomatous herb that is primarily distributed in the desert grasslands of northwestern China, especially in the Inner Mongolian Plateau, the Hexi Corridor of Gansu, central and northern Ningxia, and Northern Shanxi (Ma, 1994). It also occurs in the Gobi Desert of Mongolia, especially in Ömnögovǐ and Bayankhongor Provinces in the south and southwest of the country (Hilbig, 1995). *Psammochloa villosa* is ecologically widespread at low and high elevations (900–2,900 m) (Wu & Phillips, 2006). Its flowering and fruiting period is from September to November, and the seeds are 5–7 mm long with an average weight of 5.507 ± 0.053 mg (mean ± standard error; Huang, 2003). These lightweight seeds are potentially dispersed by the high winds that occur throughout much of its natural desert habitat. Nevertheless, seedlings are rarely observed (Zhu et al., 2005). *Psammochloa villosa* is known to have high resistance to drought, cold, alkaline soils, disease, wind, and burial by sand, all of which likely represent evolutionary adaptations that facilitate its survival in grassland and dune areas (Lu, 1987; Wu & Phillips, 2006). Previous research on *P. villosa* has been mainly focused on studying its anatomy, embryology, and microbiology (e.g., Huang et al., 2004; Lv et al., 2018; Wang et al., 2011), with only a few focused on molecular markers (e.g., Li & Ge, 2001).

In this study, we investigated the influence of aridification and climatic oscillations on the genetic structure and evolutionary processes of *P. villosa* during the Quaternary in northwestern China using a population genetics approach based on amplified fragment length polymorphism (AFLP) combined with ecological niche modeling (ENM) to compare past, present, and future environmentally suitable habitats for the species. We used AFLPs, which were multilocus markers, and their mode of inheritance was dominant, because they remained extremely efficient for investigating genetic diversity, genetic structure, and population demography due to their high levels of polymorphism, their reproducible, reliable results that were unaffected by the developmental stage of plant materials, and their universality among plant species (Wang et al., 2008). In addition, they have been used to resolve genetic structures and population demography in many diverse grass species such as *Oryza sativa*, *Leymus racemosus*, *Orinus thoroldii,* and *O. kokonoricus* (Cai et al., 2017; Liu et al., 2019; Zhang & Jia, 2002). Our main objectives were to (1) analyze the genetic structure of *P. villosa* from the Inner Mongolian Plateau using an AFLP dataset representing 43 populations, (2) test whether historical genetic divergence occurred among populations in response to Quaternary climate oscillations, and (3) evaluate the abiotic factors that are most influential in driving the distributions of *P. villosa* through time. Moreover, because no assessment of the conservation needs of *P. villosa* had previously been accomplished, we performed a preliminary assessment based on extent of occurrence (EOO) with interpretation according to guidelines of the International Union for the Conservation of Nature (IUCN). We believe that, taken together, our results can provide a scientific basis for improved protection and sustainable utilization (e.g., as forage) of *P. villosa* within the fragile desert grassland ecosystems where the species occurs.

## 2 MATERIALS AND METHODS

### 2.1 Population sampling

We randomly sampled five to ten individuals from 43 populations of *P. villosa* in the field throughout its natural range in China and obtained a total of 210 individuals (Table S1 & Figure S1). We sampled individuals spaced at least 20 m apart in order to avoid sampling a single clone more than once. In the field, we immediately put the fresh leaves into sealed bags filled with silica gel and then stored them in the laboratory in a −20℃ freezer until processing. At each population collection locality, we obtained geocoordinates using a GPS measuring instrument (Garmin eTrex201x). From each population, we obtained and deposited one representative voucher specimen in the herbarium of Qinghai‐Tibet Plateau Museum of Biology (QTPMB), Northwest Institute of Plateau Biology, Chinese Academy of Sciences, China.

### 2.2 DNA extraction and AFLP scoring

We extracted total DNA from each sample according to a modification of the CTAB procedure (Doyle & Doyle, 1987) and accessed DNA quality using 1.0% agarose gel electrophoresis and the A260/A280 ratio determined on a Nanodrop 2000c. Our procedure to obtain AFLPs was based on a modification of the method in Vos et al. (1995), and we used the restriction enzymes *EcoR*I/*Mse*I and a combination of eight primers (AAC/CAA, AAG/CAC, ACA/CAG, ACT/CAT, ACC/CTA, ACG/CTC, AGC/CTG, and AGG/CTT). We separated the fluorescently labeled fragments on an ABI PRISM 377 DNA Calibrator (Applied Biosystems) with an internal size standard, allowing visual inspection of all individual sites. As in Liu et al. (2019), we read the data every 2 bp using GeneScan ROX‐500 and set the internal standard range from 70 to 500. This approach enabled us to produce a matrix by comparing the position of the molecular weight internal standard (LIZ‐500) in each lane with the position of the peak of each sample. Subsequently, we recorded each band (monomorphic or polymorphic) in a dominant manner and transformed into either a 0 (absent) or 1 (present) matrix based on interpretations from GeneScan 3.1 (Applied Biosystems). Only bands scored unequivocally were included in the analysis (Rocha et al., 2015). In assessing the recovered fragments, we did not account for polyploidy because *P. villosa* was known to be diploid (2n = 40; Li et al., 2012). In total, we assessed 1,728 AFLP markers for the 210 individuals, and all interpretations were performed randomly. Besides, we estimated the error rate with the ratio between the observed number of phenotypic differences and the total number of phenotypic comparisons in order to track and assess genotyping errors (Bonin et al., 2010).

### 2.3 Genetic diversity and population genetic structure

For each population, we evaluated genetic diversity and population genetic structure according to standard metrics GenAlEx 6.5 (Peakall & Smouse, 2012) and AFLP‐SURV v1.0 under the assumption of Hardy–Weinberg equilibrium (HWE, Vekemans et al., 2002). These metrics included the number of individuals (*N*), percentage of polymorphic loci (PPL), observed number of alleles (*N*a), effective number of alleles (*Ne*), Shannon's information index (*I*; Lewontin, 1972), Nei's genetic diversity (*h*), expected heterozygosity (*H*e), Nei's standard genetic distance (GD), total population diversity (*H*t), genetic diversity within populations (*H*s), genetic diversity between populations (*H*b), and the population differentiation (*F*_ST_). Meanwhile, we inferred the correlation between *I* and *h*, and *I* and *H*e using spearman ranking in R 4.04 (http://www.r‐project.org/), and calculated the degree of genetic differentiation between populations (*G*_ST_) as (*H*t − *H*s)/*H*t (Nei, 1973), the parameter of gene exchange as *N*m = 0.5(1 − *G*_ST_)/*G*_ST_ (McDermott & McDonald, 1993), genetic diversity, coefficients of gene differentiation, and gene flow for eight pairs of AFLP primers in POPGENE 1.32 (Yeh et al., 1999). In addition, to eliminate the influence of codominance from AFLP molecular markers, we estimated the population differentiation (*θ* ^B^) using the Bayesian method in HICKORY v1.1 (Holsinger & Lewis, 2007; Holsinger et al., 2002), whose advantages of this method were that it did not assume HWE. We performed the Full, *f* = 0, and *θ* ^B^ = 0 models with default parameters (burn‐in = 5,000, sample = 25,000, thin = 5) and determined the most suitable model based on the deviation information criterion (DIC) (Holsinger & Wallace, 2004).

Identifying the genetic structure of species or clusters of genetically associated populations could facilitate the detection of finer‐scale geographic structures (i.e., within groups) juxtaposed with broader, regional patterns (i.e., between groups) (Li et al., 2020). To assess structuring among the 43 populations of *P. villosa*, we generated a UPGMA tree from the genetic distance matrix derived from the binary AFLP dataset and performed bootstrapping of this tree using a custom R script (Supplemental File 1). We used the UPGMA tree to identify strongly supported clusters of populations. As complementary to the UPGMA approach, we also constructed a similarity‐based network in SplitsTree 4.13 (Huson & Bryant, 2006) to infer the relationships between individuals and populations by applying the Neighbor‐Net algorithm with Jaccard's measure of distance. We further examined clusters of populations using a principal coordinate analysis (PCoA), from which we determined the optimal number of clusters by calculating the gap statistic (Tibshirani & Hastie, 2001) for axes one and two. The gap statistic represents a mathematically tractable method compared to the examination of a scree plot (Thorndike, 1953). In addition, we evaluated clusters of populations using SAMOVA, which took geographic adjacency into account. Within SAMOVA, we used a *K*‐means method to select the best clustering scheme based on genetic variation coefficients (*F*_CT_) (Li et al., 2020). For possible numbers of clusters, *K*, in the range two to ten, we performed 100 heuristic searches with 10,000 steps each, and we selected the value of *K* that minimized within‐cluster *F*_CT_ without over‐partitioning. Subsequently, we inferred clusters of populations of *P. villosa* using STRUCTURE V2.2 (Hubisz et al., 2009), which differed from SAMOVA by not requiring that groupings be geographically adjacent. In STRUCTURE, we performed the analyses using an admixture model with independent allele frequencies for 90 independent runs for the number of clusters (*K*) ranging from one to ten. We applied 2 × 10^5^ repetitions of the Markov chain Monte Carlo with a burn‐in of 25%. To determine the best value of *K* for the STRUCTURE analyses, we used the Δ*K* statistical method (Evanno et al., 2005). Based on all assessments on the optimal number of clusters of populations and the constituent populations of the optimal grouping, we further evaluated the genetic variation between populations within groups and between groups in SAMOVA 1.0 via an analysis of molecular variance (AMOVA, Excoffier et al., 1992) in ARLEQUIN v3.01 (Excoffier et al., 2005). Moreover, we performed the tests of neutrality with Tajima's *D* and Fu's *F*s in ARLEQUIN v3.01 (Excoffier et al., 2005) and determined the correlation between *F*_ST_ inferred from the binary matrix of scored AFLPs and geographic distance of the populations via a Mantel test (Mantel, 1967) in GenAlEx 6.5 (Peakall & Smouse, 2012) with 9,999 permutations to evaluate significance.

### 2.4 Distribution modeling of *P. villosa*

In order to predict the effects of Quaternary climatic oscillations on the geographic distributions of *P. villosa*, we used ecological niche modeling (ENM) to compare the potential distributions of *P. villosa* at the Last Inter‐Glacial (LIG, ~120,000–140,000 years before present), the Last Glacial Maximum (LGM, ~21,000 years before present), and the present. As input for the ENMs, we used the geocoordinates of the 43 populations of *P. villosa* that we sampled for this study as well as geocoordinates of specimens recorded in the Chinese Virtual Herbarium (CVH, http://www.cvh.ac.cn), the Global Biodiversity Information Facility (http://www.gbif.org), the China National Specimen Information Infrastructure (http://www.nsii.org.cn), and the Specimen Resources Sharing Platform for Education (http://mnh.scu.edu.cn/main.aspx). In total, after removing duplicate and ambiguous records, we obtained 155 georeferenced data points for using in the ENMs (Table S2).

Initially, to perform ENMs, we obtained 19 bioclimatic variables and three geographic factors (altitude, slope, and aspect) as global information system (GIS) layers from the WorldClim database (Hijmans et al., 2005, www.worldclim.org) at 2.5 arc‐min resolution. This represented 22 total environmental variables for modeling. We followed Peterson and Nakazawa (2008) and applied the Spearman's correlation test to exclude highly correlated variables, a correlation of <0.75 compared to other variables. We performed preliminary modeling in MaxEnt 3.3.3k (Phillips & Dudík, 2008) with 75% of localities randomly selected for training and 25% selected for testing 500 times independently to ensure reliable results. We used these models to assess the relative contributions of the 22 environmental variables and excluded those exhibiting a relative contribution score ≥0.8.

Based on the outcome of Spearman's and the preliminary ENMs, we retained 11 variables to generate the final models. We preformed modeling using the same procedure as above with these 11 variables and the occurrence data and subsequently evaluated model performance using the area under the curve (AUC) of the receiver operating characteristic (ROC). The value of AUC ranges between 0 (randomness) and 1 (exact match), and values above 0.9 are generally regarded as indicating good model performance (Swets, 1988).

In order to obtain the geographic distribution, we projected the ENMs onto a map representing the Inner Mongolian Plateau using ArcGIS 10.2. For visualization, we divided suitable habitat into four classes according to the probability of occurrence (*p*) based on the model results: highly suitable habitat (0.5 ≤ *p* ≤ 1.0), moderately suitable habitat (0.3 ≤ *p* < .5), poorly suited habitat (0.1 ≤ *p* < .3), and unsuitable habitat (0.0 ≤ *p* < .1). We performed projections based on all time periods: LIG, LGM, and the present. Additionally, in order to better assess the conservation needs of this species, we also projected the model into future times: the 2050s and 2070s. The data underlying the maps for future projections comprised inference of the effects of climate forcing factors (RCP8.5 and RCP2.6) according to the CCSM4 model (Van Vuuren et al., 2011).

We used the ENMs to assess niche similarity between populations occurring in the clusters, or groups, that we inferred. To accomplish this, we calculated Schoener's *D* (Schoener, 1968) and standardized Hellinger distance (calculated as *I*) in ENMTools 1.3 (Warren et al., 2010). We obtained the null distribution of niche models for the identity test based on 1,000 pseudo‐replicates generated by random sampling from the data points pooled for each pair of clusters. We determined *D* and *I* by comparing with null distributions drawn from pooled occurrences, retaining the original cluster sizes, and we generated histograms of frequency distributions in R 4.04.

### 2.5 Conservation assessment of *P. villosa*

We performed a conservation assessment for *P. villosa* using the extent of occurrence (EOO) (Moat, 2007; Velzen & Wieringa, 2014) and following guidelines for interpretation from IUCN (2001). EOO comprises the minimum convex polygon covering all known or predicted sites for the species. It is frequently used as a preliminary assessment tool, such as when a new species is described or when populations of a species are found in new places or were locally extirpated (e.g., Lachenaud et al., 2013; Velzen & Wieringa, 2014). In the case of *P. villosa*, there has been no prior conservation assessment for the species, and its status is not presently included in the IUCN Red List of Threatened Species (IUCN, 2020). Therefore, we analyzed the EOO based on the 155 occurrence data points also used for ENM (Table S3).

## 3 RESULTS

### 3.1 Polymorphism of AFLP markers

The 43 populations of *P. villosa* were all assigned correctly to the corresponding individual after amplification and scoring. The eight primer pairs yielded 1,728 clearly identifiable amplified bands, of which 1,654 (95.7%) were polymorphic (Table 1), and these differences were observed for 71,122 phenotypic comparisons, giving an error rate of 2.3%. Subsequently, different primers yielded different numbers of bands ranging from 214 (*E*‐ACG/*M*‐CTC) to 199 (*E*‐AGC/*M*‐CTG and *E*‐AAG/*M*‐CAC) with an average of 207. The highest rate of polymorphism for an individual primer was 99.1%, and the lowest was 92.1%. Overall, these eight primers showed high levels of polymorphism among individuals of *P. villosa*.

**TABLE 1:** Summary statistics for eight selective primer combinations of amplified fragment length polymorphism (AFLP) in the present study

Selective nuclear	Polymorphism band	Amplification band	PPL (%)	Size range (bp)	*N*a	*N*e	*h*	*I*	*H*t	*H*s	*G*_ST_	*N*m
E‐AAC/M‐CAA	201	216	93.1	69.5–501.5	2.00	1.34	0.21	0.34	0.210	0.117	0.442	0.633
E‐AAG/M‐CAC	199	216	92.1	69.5–501.5	2.00	1.33	0.20	0.33	0.203	0.113	0.446	0.622
E‐ACA/M‐CAG	206	216	95.4	69.5–501.5	2.00	1.32	0.21	0.34	0.207	0.113	0.453	0.603
E‐ACT/M‐CAT	211	216	97.7	69.5–501.5	2.00	1.33	0.21	0.34	0.207	0.090	0.565	0.386
E‐ACC/M‐CTA	211	216	97.7	69.5–501.5	2.00	1.31	0.20	0.32	0.199	0.112	0.437	0.644
E‐ACG/M‐CTC	214	216	99.1	69.5–501.5	2.00	1.29	0.19	0.32	0.192	0.113	0.411	0.716
E‐AGC/M‐CTG	199	216	92.1	69.5–501.5	2.00	1.34	0.21	0.34	0.213	0.108	0.496	0.509
E‐AGG/M‐CTT	213	216	98.6	69.5–501.5	2.00	1.33	0.21	0.34	0.209	0.104	0.502	0.496
Total	1,654	1,728	‐	‐	‐	‐		‐	‐	‐		‐
Average	207	216	95.7	‐	2.00	1.32	0.21	0.33	0.205	0.109	0.469	0.576

Abbreviations: *G*_ST_, the genetic differentiation between populations; *h*, Nei's genetic diversity; *H*s, the average gene diversity within populations; *H*t, total gene diversity; *I*, Shannon's information index; *N*a, observed number of alleles; *N*e, effective number of alleles; *N*m, gene flow; PPL, percentage of polymorphic loci.

Our measurements of the genetic diversity indices based on the eight primer pairs revealed that average *N*a was 2.00 for each primer, while *N*e ranged from 1.29 to 1.34 with an average of 1.32, *h* ranged from 0.19 to 0.21 with an average of 0.21, and *I* ranged from 0.32 to 0.34 with an average of 0.33. The primer pair *E*‐AGG/*M*‐CAC exhibited the highest diversity based on these indices, while *E*‐ACT/*M*‐CTT had the lowest. In general, each of eight primer pairs appeared to facilitate a robust assessment of genetic diversity in *P. villosa*.

### 3.2 Clusters of populations and genetic structuring

**FIGURE 1:**
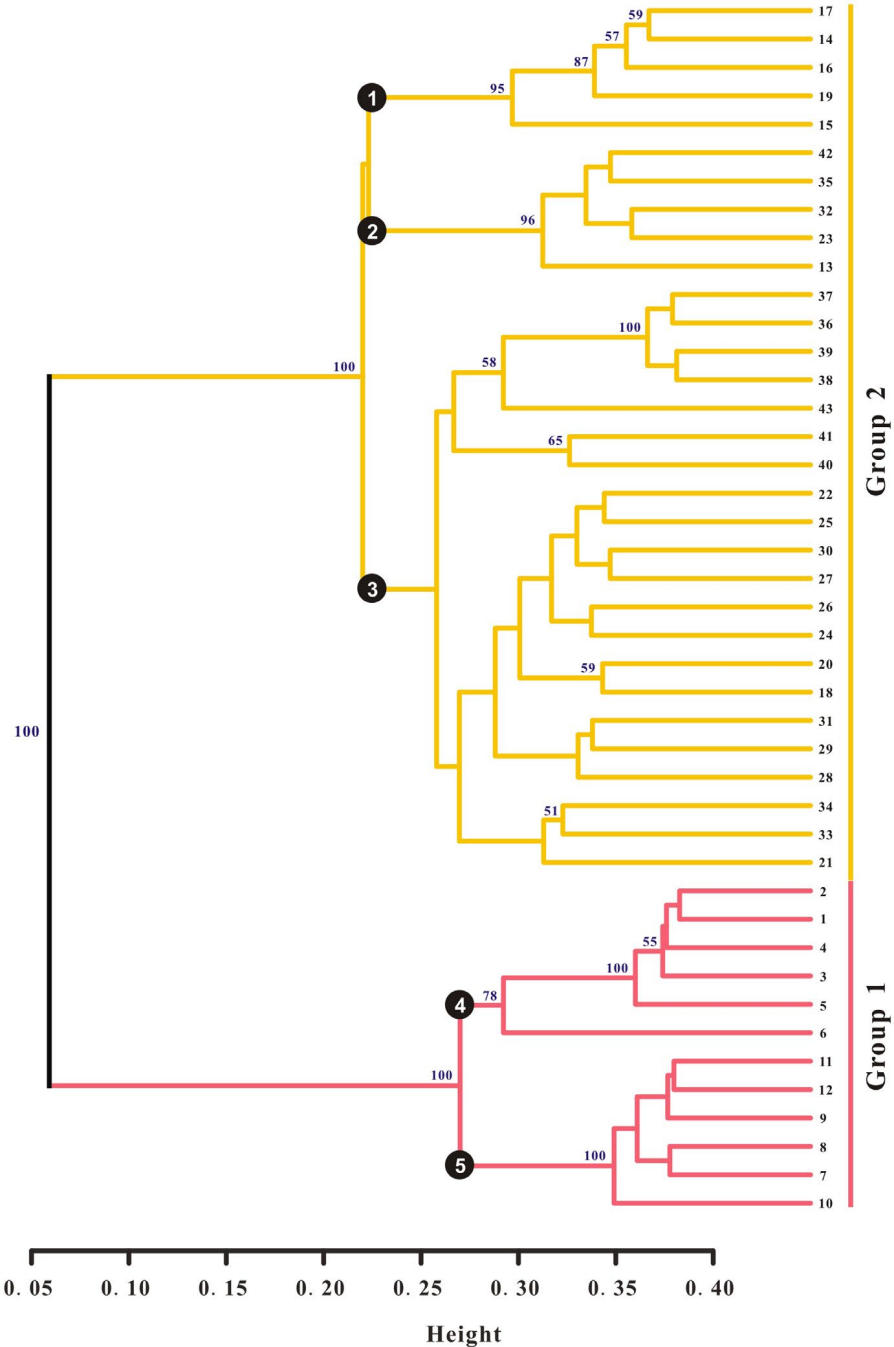
Dendrogram of *P. villosa* generated by unweighted pair group method analysis (UPGMA) cluster analysis from the genetic similarity matrix obtained using amplified fragment length polymorphism genetic distance (see Figure S1 for population codes)

**FIGURE 2:**
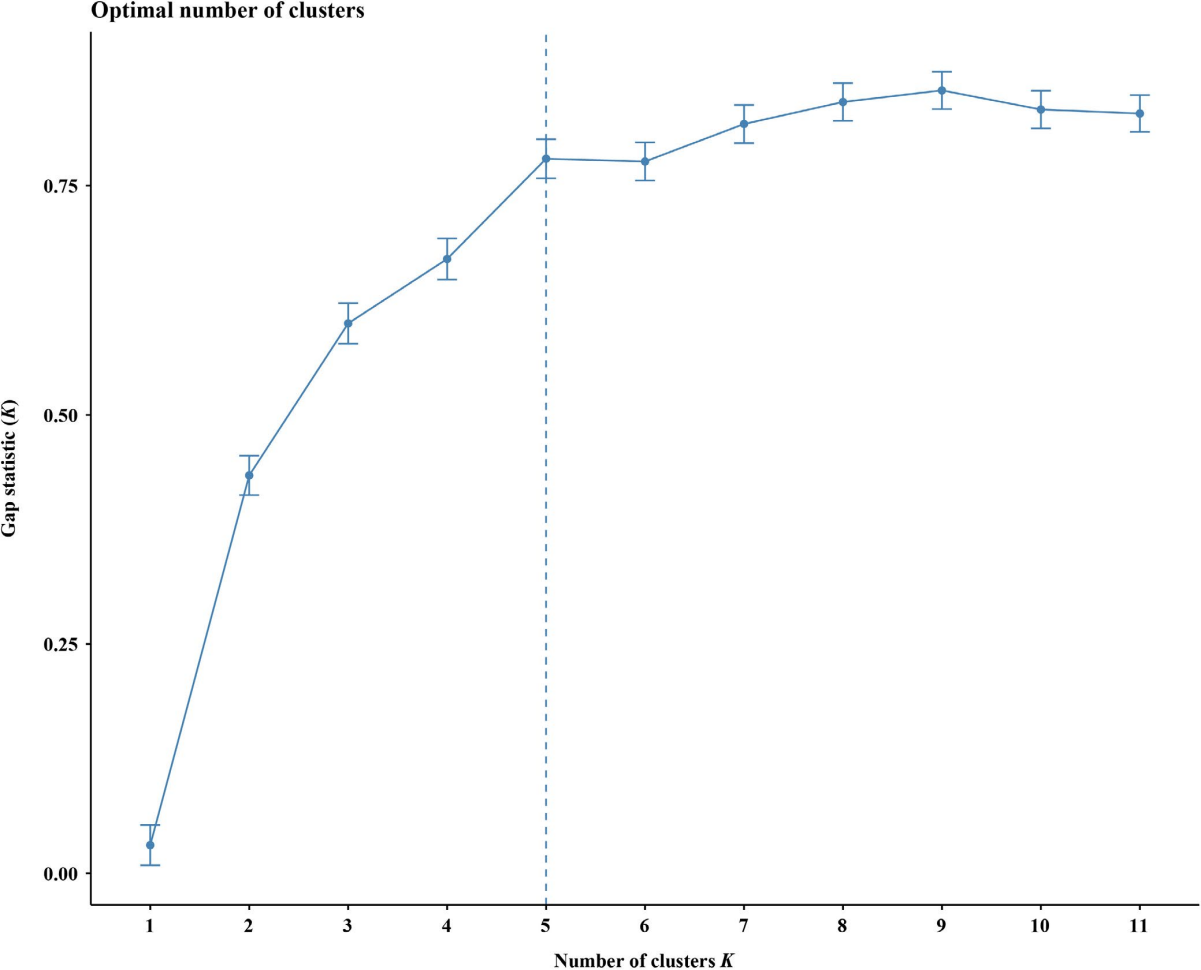
The gap statistical analysis based on all 210 individuals of *P. villosa*. The dotted line represents the optimal number of clusters

**FIGURE 3:**
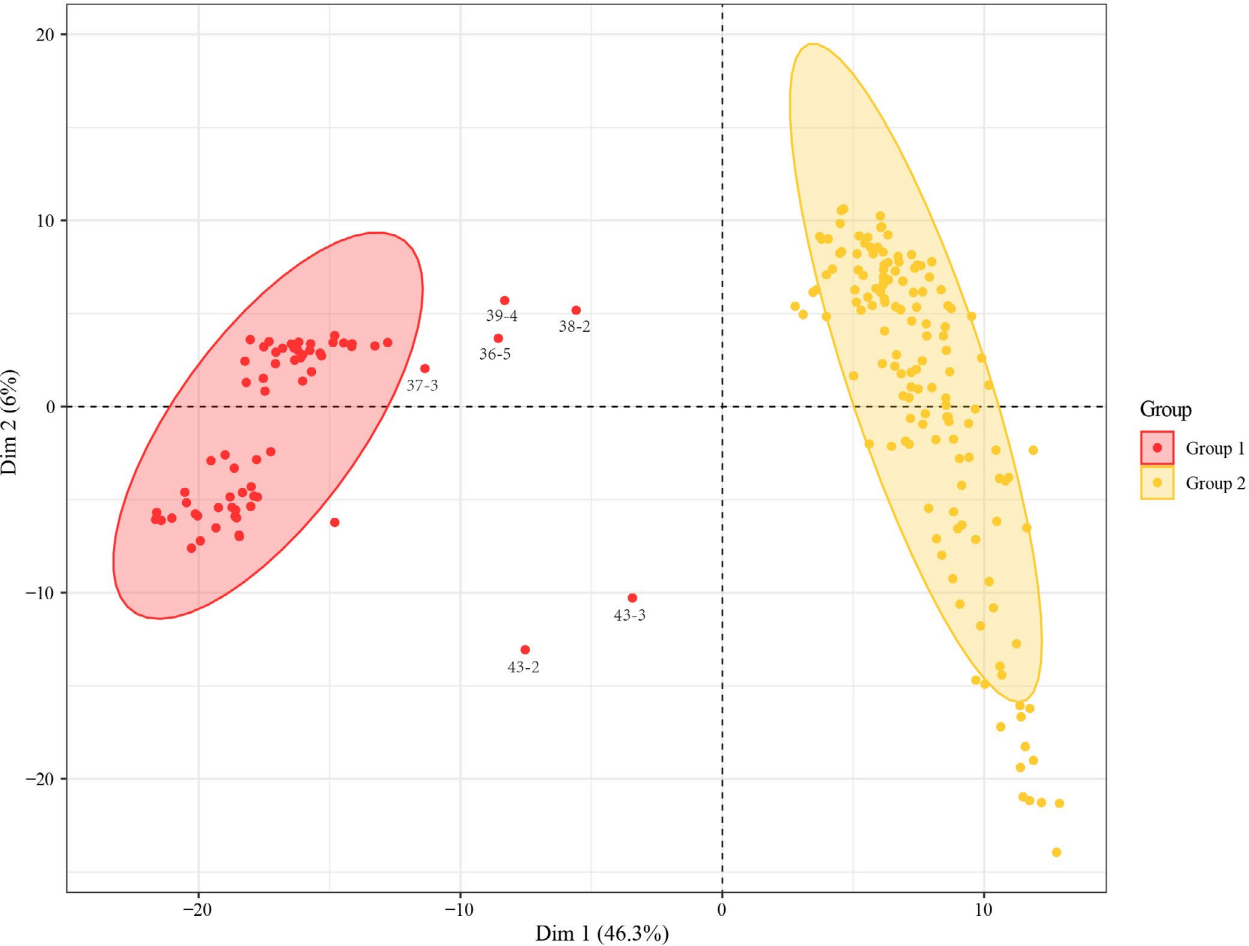
A two‐dimensional plot of the principal coordinate analysis (PCoA) based on variation of amplified fragment length polymorphism markers for *P. villosa* (see Figure S1 for population codes; ellipse, 95% confidence interval)

**FIGURE 4:**
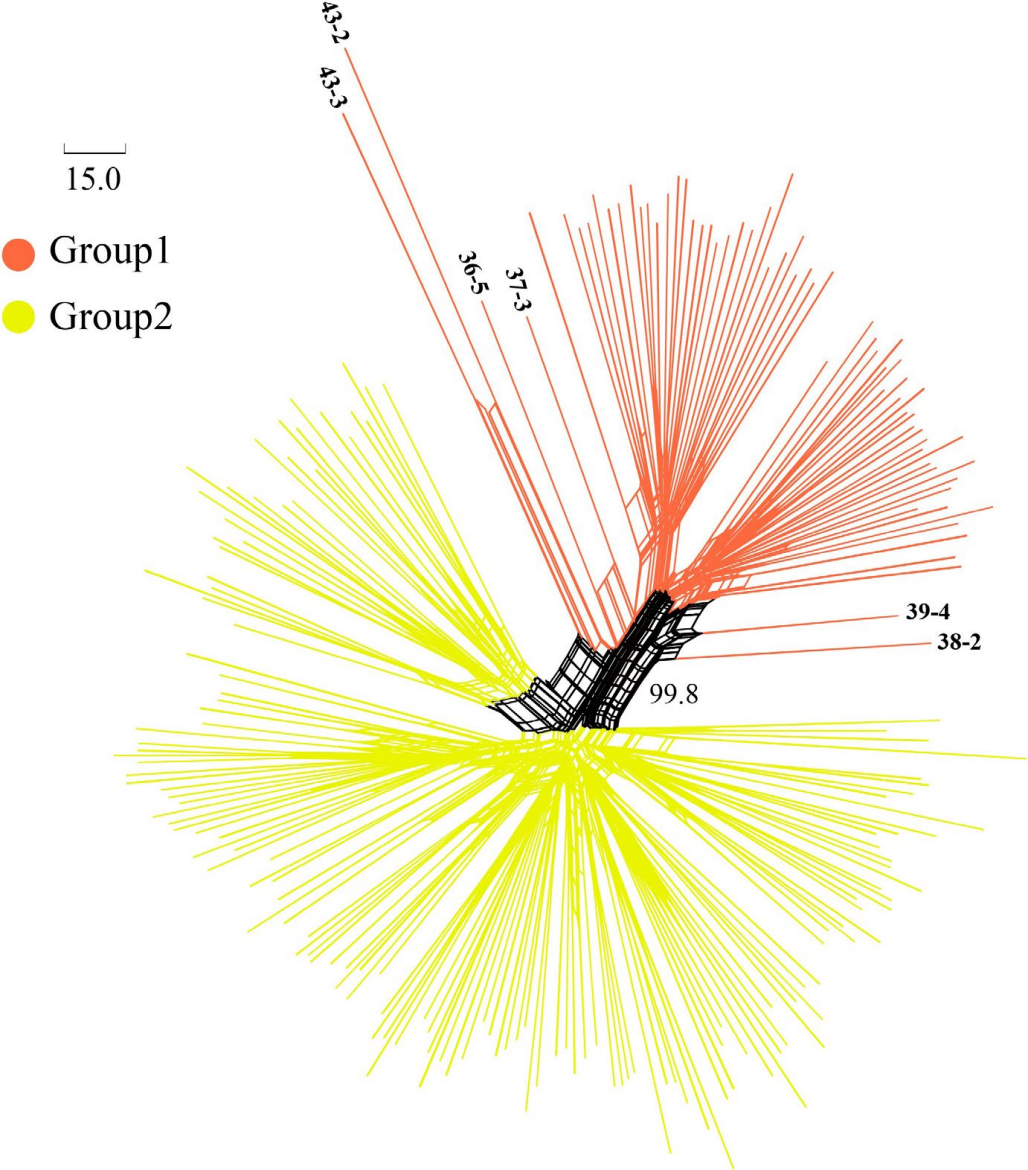
Neighbor‐Net split network of *P. villosa* based on amplified fragment length polymorphism datasets using Jaccard's distances. Lines of red and yellow represent Group 1 and Group 2, respectively

**FIGURE 5:**
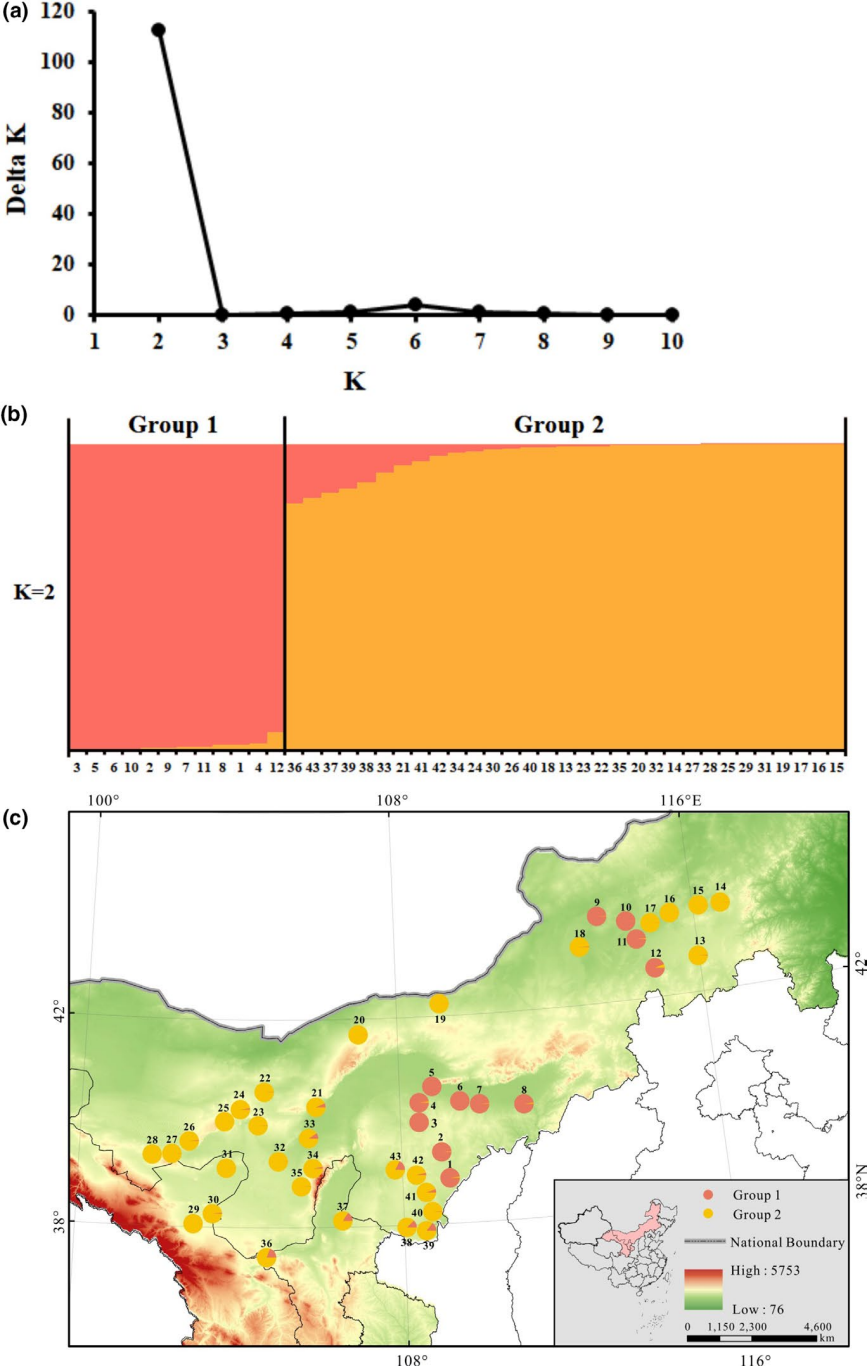
Results of the Bayesian clustering analysis in STRUCTURE of 210 individuals representing *P. villosa*. (a) Δ*K* values from the mean log‐likelihood probabilities through STRUCTURE runs where inferred cluster (*K*) ranged from one to ten; (b) Estimated genetic clustering for *K* = 2, where unique colors correspond to assignment at different clusters; (c) Geographic origin from 43 populations of *P. villosa* and their color‐coded grouping according to the structure analysis for the model with *K* = 2

The UPGMA analysis on 43 populations of *P. villosa* showed that these populations could be assigned to two clusters that have high support (100% bootstrap support (BS), Figure 1). The gap statistical analysis based on all 210 individuals yielded five groups in which individuals generally clustered within their populations (Figure 2), and groups of populations represented subgroups on the UPGMA tree. Based on SAMOVA, we found that the optimal number of clusters of populations was three (*K* = 3) (Table S3). As with the PCoA analysis (Figure 3), the three groups represented subgroupings of the two highly supported ones according to UPGMA. Specifically, separate groups of populations (P) 1‐6 and P7‐12 were resolved in SAMOVA. However, in the field, we observed striking similarities in habitat among P6‐8, which were also geographically proximal. Meanwhile, both SplitsTree and STRUCTURE revealed two clusters of populations (Figures 4 and 5). However, in the SplitsTree analysis, some individuals from populations 36, 37, 38, 39 did not cluster with others from their populations and were resolved in the opposing cluster (Figure 4). These same individuals also showed similar phenomenon in the analysis of PCoA. Overall, we chose to treat the populations as belonging to the two groups identified based on UPGMA, though we acknowledged that results from other analyses, such as the gap statistic and SAMOVA, suggested that additional structures with weaker signal might also exist among the sampled populations.

Hereafter, we refer to the two groups as Groups 1 and 2, which comprise P1‐12 and P13‐43, respectively. Populations of Group 1 occurred mainly in the central and eastern regions of the Inner Mongolian Plateau, while populations of Group 2 were distributed throughout the range of the species in China. Notably, the populations of Group 1 tended to be found at lower elevations compared to those of Group 2 (Table S1 & Figure S1).

### 3.3 Genetic diversity of populations

The percentage of polymorphic loci (PPL), observed number of alleles (*N*a), effective number of alleles (*N*e), Shannon information index (*I*), Nei's genetic diversity (*h*), and expected heterozygosity (*H*e) of 43 populations were from 25.7 to 54.8, 0.50 to 1.12, 1.19 to 1.38, 0.13 to 0.32, 0.10 to 0.22, and 0.13 to 0.22, respectively (Table S1). The PPL, *N*a, *N*e, *I*, and *h* of populations from Group 1 were 37.6, 0.72, 1.22, 0.19, and 0.13, respectively, while those of Group 2 were 39.5, 0.73, 1.21, 0.18, and 0.13 (Table 2). The genetic diversity showed that the populations of Group 1 exhibited minimally greater genetic diversity than those of Group 2 (except the value of *N*a). In addition, we noted a strong correlation between *I* and *h* (Table 1: Spearman ranking correlation, *R* = 0.976, *p* = 0; Table S1: Spearman ranking correlation, *R* = 0.973, *p* = 0) and between *I* and *H*e (Table S1: Spearman ranking correlation, *R* = 0.885, *p* = 0). Thus, hereafter, we use only *h* and *H*e to discuss the genetic diversity of *P. villosa*.

**TABLE 2:** Genetic diversity, differentiation parameters, and neutrality test for 43 populations of *P. villosa* in the present study

Population group	Populations of group 1 (P1‐12)	Populations of group 2 (P13‐43)	All populations
Polymorphic loci (%)	37.6	39.5	39.0
Observed number of alleles (*N*a, *SE*)	0.72 (0.007)	0.73 (0.004)	0.73 (0.019)
Effective number of alleles (*N*e, *SE*)	1.22 (0.002)	1.21 (0.001)	1.22 (0.006)
Shannon's information index (*I*, *SE*)	0.19 (0.002)	0.18 (0.001)	0.19 (0.005)
Nei's genetic diversity (*h*, *SE*)	0.13 (0.001)	0.13 (0.001)	0.13 (0.004)
Expected heterozygosity (*H*e, *SE*)	0.20 (0.004)	0.20 (0.004)	0.20 (0.005)
Total gene diversity (*H*t)	0.179	0.200	0.206
Gene diversity within populations (*H*s, *SE*)	0.158 (0.002)	0.165 (0.003)	0.162 (0.002)
Genetic diversity between populations (*H*b, *SE*)	0.021 (0.000)	0.035 (0.002)	0.043 (0.002)
Genetic differentiation between populations (*G*_ST_)	0.340	0.435	0.469
Gene flow (*N*m)	1.000	0.661	0.576
Population differentiation (*F*_ST_)	0.115*	0.177*	0.211*
Population differentiation (*θ* ^B^)	0.199	0.265	0.314
Fu's *Fs* (*p*)	3.249 (0.570)	3.358 (0.574)	3.327 (0.570)
Tajima's *D* (*p*)	0.113 (0.589)	0.059 (0.581)	0.074 (0.580)

Abbreviation: *SE*, standard error.

* Significant at *p* < .001.

Across all 43 populations of *P. villosa*, *H*t was 0.206, *H*s was 0.162, *H*b was 0.043, *F*_ST_ was 0.211, *G*_ST_ was 0.469, *θ* ^B^ was 0.314, and *N*m was 0.576, indicating a limited level of genetic exchange within the species. In Group 1, *H*t = 0.179, *H*s = 0.158, *H*b = 0.021, *F*_ST_ = 0.115, *G*_ST_ = 0.340, *θ* ^B^ = 0.199, and *N*m = 1.000 (Table 2). This differed from Group 2, which exhibited higher total genetic diversity (*H*t = 0.200), genetic diversity within populations (*H*s = 0.165), genetic diversity between populations (*H*b = 0.035), the population differentiation (*F*_ST_ = 0.177; *θ* ^B^ = 0.265), differentiation among populations (*G*_ST_ = 0.435), and historic gene flow (*N*m = 0.661). Meanwhile, we found that the value of DIC for the Full model was the lowest, both the population level and group level (Table S4), representing the most suitable model, and the genetic differentiation between populations of Group1 (*θ* ^B^ = 0.199) or Group2 (*θ* ^B^ = 0.265) was lower than those of all populations (*θ* ^B^ = 0.314). It was important to note that the genetic differentiation of Group2 was greater than that of Group1, which indicated that the genetic structure and genetic diversity of *P. villosa* would not be impacted by the consideration of HWE.

Analysis of molecular variance (AMOVA) based on the 43 populations showed that the proportion of genetic variation among populations was lower (35.8%, *F*_ST_ = 0.358, *p* < .001) than within populations (64.2%) (Table 3). When aggregating populations of Groups 1 and 2, we found that 22.4% of the genetic variation occurred among populations within groups (*F*_CT_ = 0.285, *p* < .001), while most of genetic variation (56.1%) existed within populations (*F*_ST_ = 0.439, *p* < .001). Overall, the genetic variation at the population‐ and local geographic‐scale was much higher than regionally in *P. villosa*. Additionally, the result of the neutrality test suggested the value of Tajima's *D* and Fu's *Fs* was positive, but nonsignificant for all populations of *P. villosa* (Table 2).

**TABLE 3:** Results of analyses of molecular variance (AMOVAs) based on amplified fragment length polymorphism markers for *P. villosa*

Grouping	Source of variation	*df*	SS	VC	Percent variation (%)	Fixation index
Total populations	Among populations	42	20,586.26	73.46	35.8%	*F*_ST_ = 0.358**
	Within populations	167	21,960.47	131.50	64.2%	
	Total	209	42,546.72	204.96		
UPGMA groups	Among groups	1	4,707.10	50.34 Va	21.5%	*F*_CT_ = 0.215**
	Among populations within groups	41	15,879.16	52.42 Vb	22.4%	*F*_SC_ = 0.285**
	Within populations	167	21,960.47	131.50 Vc	56.1%	*F*_ST_ = 0.439**
	Total	209	42,546.72	234.26		

Abbreviations: *df*, degrees of freedom; *F*_CT_, variance among groups relative to total variance; *F*_SC_, variance among populations within groups; *F*_ST_, variance among populations; SS, sum of squares; VC, variance components.

** Significant level: *p* < .001.

**FIGURE 6:**
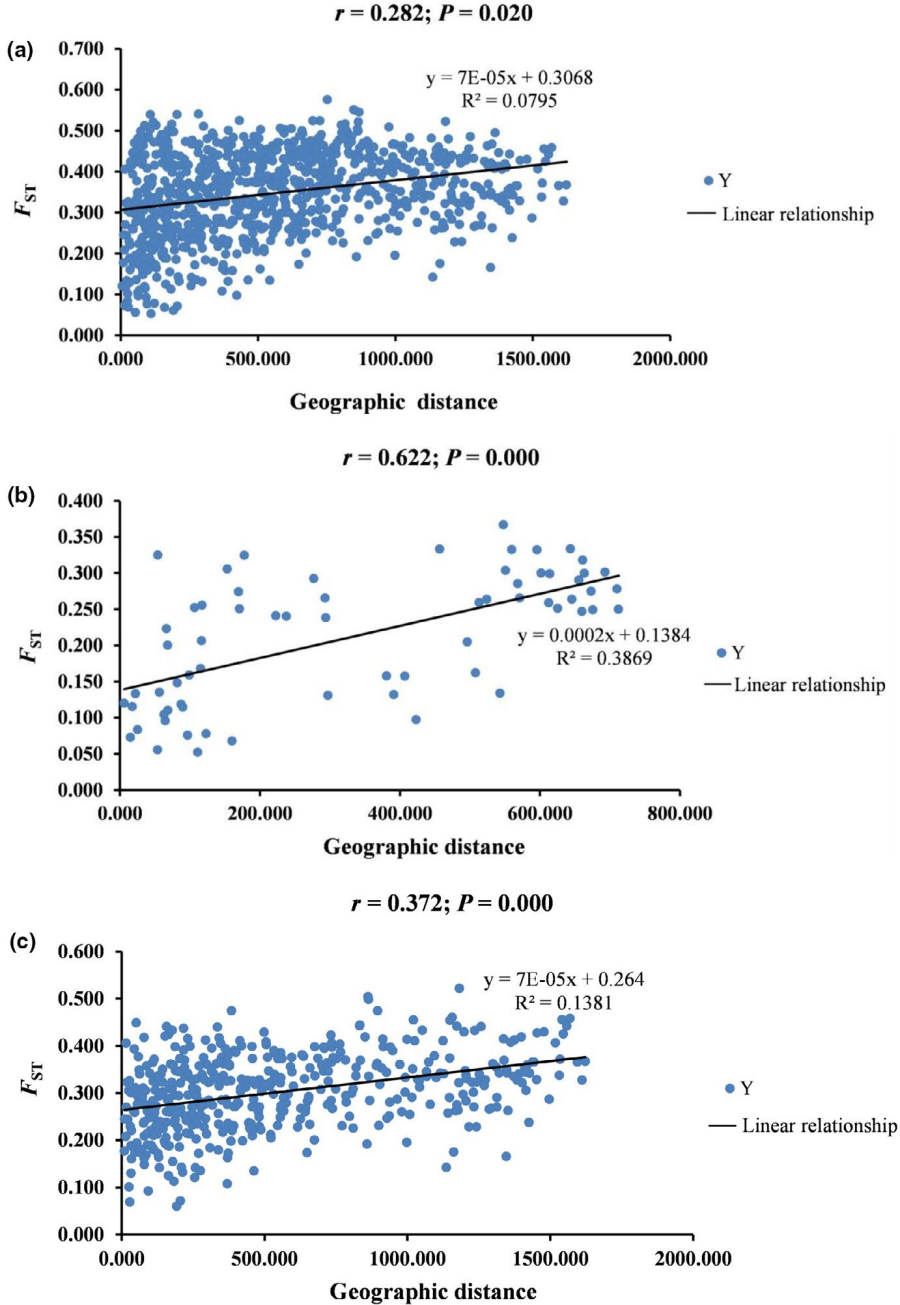
Correlation of Mantel test between geographic distance and *F*_ST_. (a) Mantel test from 43 populations of *P. villosa*; (b) Mantel test from populations of Group 1; (c) Mantel test from populations of Group 2

The Mantel test revealed that there was a significant positive correlation between geographic distance and *F*_ST_ for the 43 populations (*r* = .282, *p* < .05) (Figure 6). Similarly, we detected a strong, significant, positive correlation between geographic distance and *F*_ST_ for Group 1 (*r* = .622, *p* < .05) and a weak but significant positive correlation for Group 2 (*r* = .372, *p* < .05).

### 3.4 Distributional change of *P. villosa*

**FIGURE 7:**
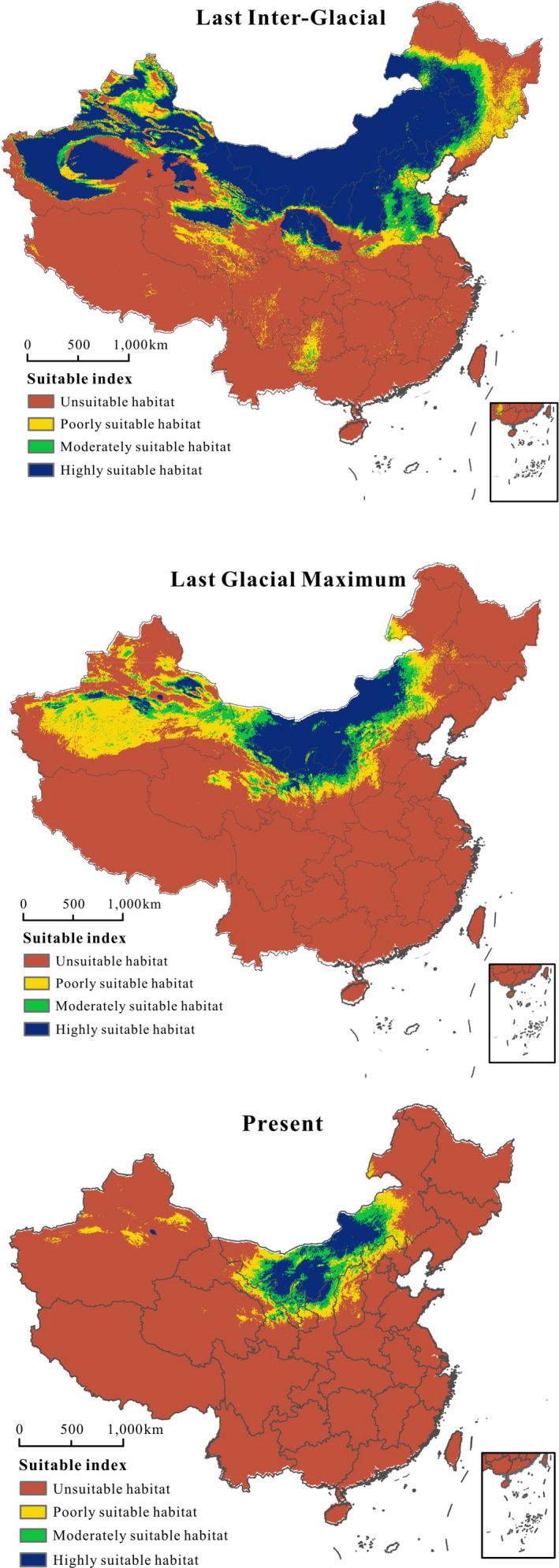
Potentially suitable climatic distribution of *P. villosa* under different climate change scenarios in the Inner Mongolian Plateau

**FIGURE 8:**
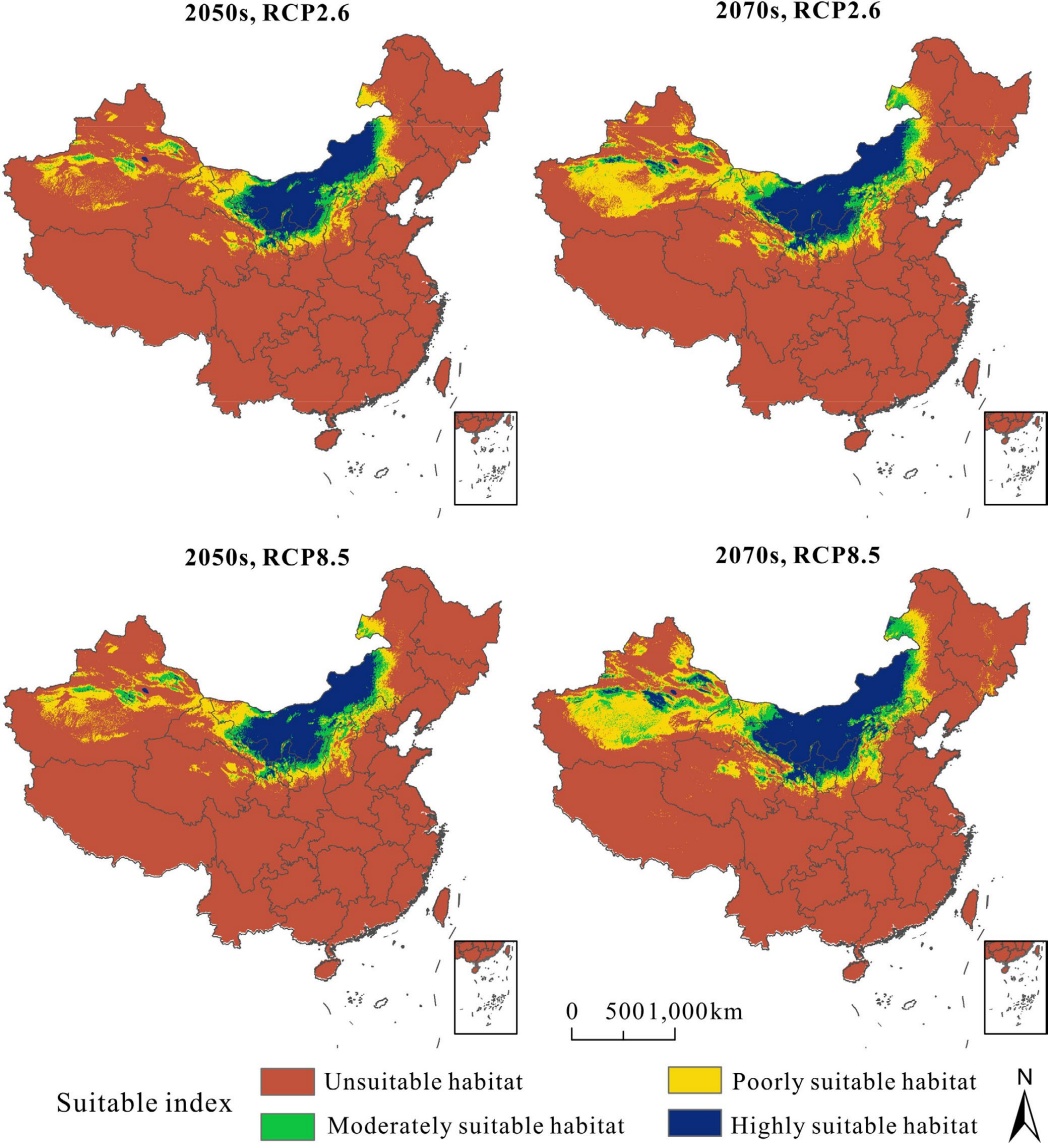
The future suitable climatic distribution of *P. villosa* in the Inner Mongolian Plateau

ENMs for *P. villosa* yielded relatively high AUC, demonstrating reliable model performance (AUC = 0.969, Figure S2). For the eleven variables used for modeling, the most significant factor for the spatial distribution pattern of *P. villosa* was the altitude (Alt), followed by temperature annual range (Bio 7) and precipitation of warmest quarter (Bio 18), whose contribution rates were 40.0%, 17.2%, and 16.7%, respectively (Table S5). Based on model projections, we observed a contraction in a highly suitable habitat during the LGM compared with the LIG (Table 4 & Figure 7). Nevertheless, there was less suitable highly suitable habitat in the present compared to during either the LIG or LGM (Figure 7). Highly suitable habitat projected for the present day was largely congruent with the actual geographic distribution of *P. villosa*, within the Inner Mongolia Plateau. Simultaneously, we estimated the future changes in the potential spatial distribution under the RCP 2.6 and RCP 8.5 scenarios for the 2050s and 2070s. According to the future model predictions, our projections of the models based on future climates showed that, in general, the areas of suitable habitat for *P. villosa* would remain stable under the climatic scenario of RCP 2.6 for the 2050s and 2070s, whereas there was an increase in highly suitable areas based on RCP 8.5 (Table 4 & Figure 8).

**TABLE 4:** Prediction of potential suitable distribution areas of *P. villosa* in different periods

Period	Prediction area (×10^4^ km^2^)				
	Unsuitable habitat	Poorly suitable habitat	Moderately suitable habitat	Highly suitable habitat	Total suitable habitat
LIG	532.4994	85.2601	54.3909	282.0408	421.6918
LGM	724.5804	111.8953	45.7149	74.2954	231.9056
Present	845.8872	43.8207	30.2673	37.1570	111.2450
2050s‐2.6	804.7547	65.9706	25.4776	60.2943	151.7425
2050s‐8.5	793.9596	72.6263	29.3144	60.5905	162.5312
2070s‐2.6	804.7547	65.9706	25.4776	60.2943	151.7425
2070s‐8.5	710.9724	112.0121	47.4650	86.0193	245.4964

**FIGURE 9:**
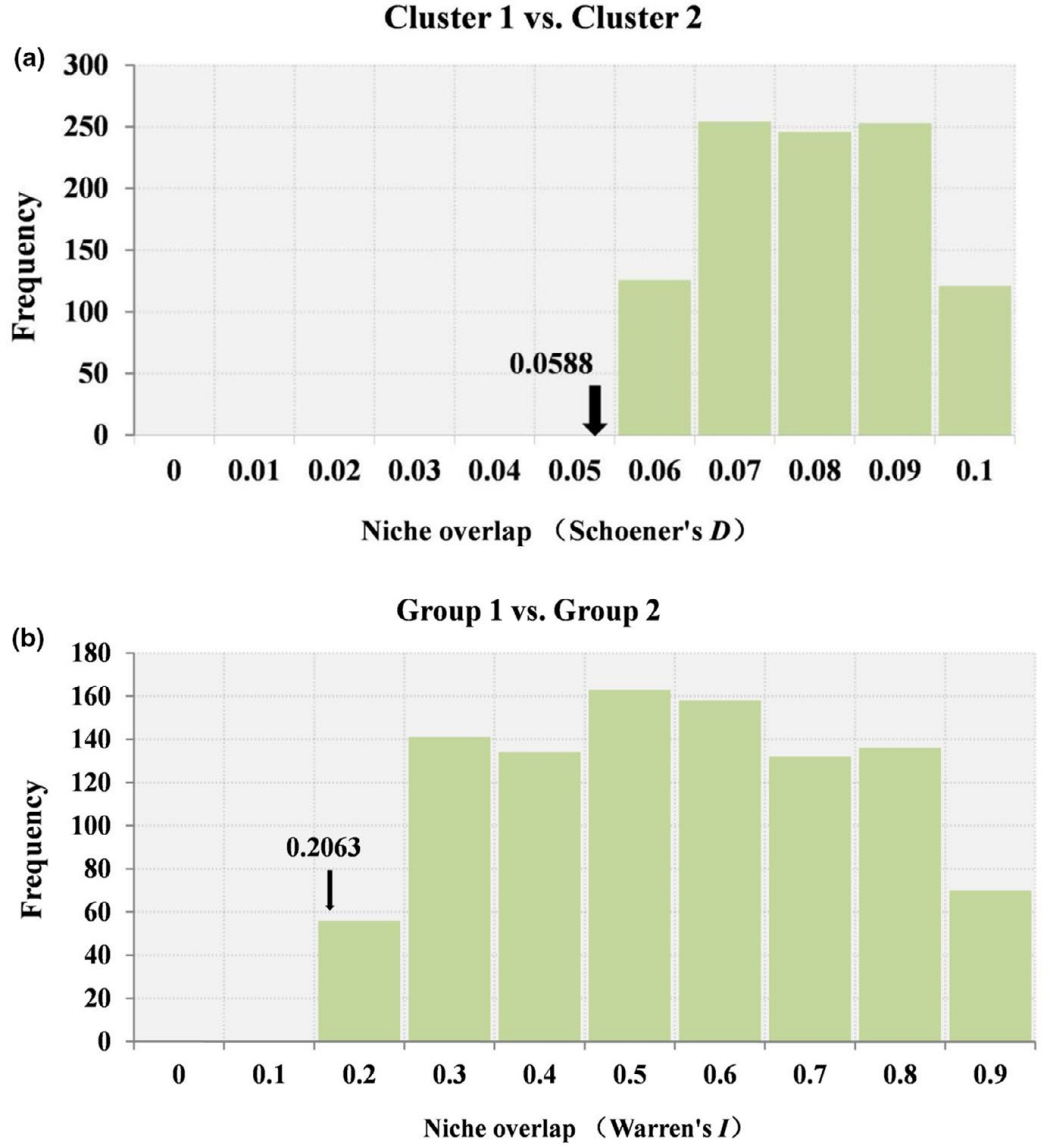
Results of the niche‐identity test. (a) Schoener's *D*; (b) Warren's *I*. The arrow in each panel represents the observed niche similarity between occurrence points for the corresponding pair of clusters. The histograms represent the distribution of niche similarities obtained from pairs of pseudo‐niches constructed by random resampling of occurrence points of the two clusters

When we compared the niches of Groups 1 and 2, we found that *D* and *I* were significantly lower than the values expected from the pseudo‐replicated datasets. Thus, there is distinct niche differentiation between the two groups (*p* < .01) (Figure 9). The niches of the two groups differed mainly in that Group 2 occurred at higher elevations and under temperature annual range.

## 4 DISCUSSION

### 4.1 Genetic diversity of *P. villosa*

Genetic diversity is closely linked to the evolutionary potential of a species to adapt to adverse environments (Ma et al., 1994). In the present study, we observed high genetic diversity at the species level in *P. villosa* (*h* = 0.21) and at the population level (*h* = 0.13). Compared to genetic diversity in other species of Poaceae assessed using AFLPs, genetic diversity in *P. villosa* was slightly lower than in *Dactylis glomerata* (*h* = 0.23; Zhang et al., 2017), which was widespread in Europe, Asia, and Africa, but higher than that of *Leymus chinensis* (*h* = 0.06), *Chascolytrum bulbosum* (*h* = 0.10), and *Leymus racemosus* (*h* = 0.19) (Cai, 2016; Gong et al., 2007; Silva et al., 2016), which also had wider distributions than *P. villosa*.

The underlying drivers of genetic diversity within species are generally a combination of biological factors, such as dispersal abilities and life history, and environmental factors, such as climate and anthropogenic activities (Gerzabek et al., 2020; Prazeres et al., 2020). The life history of *P. villosa* frequently involves clonal reproduction via its rhizomes under harsh environmental conditions, although the species also reproduce sexually by seed following wind pollination (Li & Ge, 2001; Wang et al., 1999). In comparison with *L*. *chinensis*, *C. bulbosum*, and *D*. *glomerata*, the relatively high genetic diversity of *P. villosa* might be explained by one or more factors. Among these, our study design comprised more populations, which might lead to greater accuracy in inferring genetic diversity. However, biological explanations are more likely and include possible higher clonal fitness of *P. villosa* as it has extremely robust, hardy rhizomes, and high lifetime rates of seed production and regeneration via seedlings (although annual regeneration via seedling is at a low rate) (Eriksson & Bremer, 1993; Shimizu et al., 1998).

### 4.2 Genetic differentiation and genetic structure

The genetic structure of a species is effectively the sum of genetic differentiation among and within populations (Hamrick & Godt, 1989). Overall, genetic structure occurring among populations results from the evolutionary history of the species in question; natural selection; genomic factors (e.g., mutations, reorganization, and genetic drift); and biological characteristics, including gene flow, mating system, mode of reproduction, and seed dispersal mechanisms (Slatkin, 1987; Zhen, 2010). Genetic differentiation is primarily controlled by aspects of gene flow, such as its rate and directionality (Hamrick & Godt, 1989). In plants, gene flow occurs most often via the transmission of pollen and seeds during sexual reproduction. However, for clonal species, such as *P. villosa*, asexual propagules may be more common than seeds, but these usually have limited dispersal distance and, thus, restrict gene flow among populations (Xia et al., 2002).

For *P. villosa*, we inferred that more than 56% of the genetic variation existed within populations, with an average pairwise *F*_ST_ of 0.358 for all 43 populations and gene flow (*N*m) of 0.576. According to Wright's (1978) theory, *F*_ST_ > 0.25 represents great genetic differentiation, but this can be mitigated by gene flow of *N*m > 1. In *P. villosa*, we found high *F*_ST_ but limited gene flow, which should yield high rates of differentiation among populations. However, we found higher rates of within‐population differentiation for the species. This differs from findings in other studies of *P. villosa*, such as in Li and Ge (2001), who examined seven populations, of which two were from Shihuimiao Ecological Station and five were from the Shilongmiao Ecological Station, and found higher rates of differentiation among populations. Similarly, Wang et al. (1999) assessed the genetic diversity of four populations of *P. villosa* from mobile and fixed sand dunes in the Shihuimiao Ecological Station (two populations) and the Shilongmiao Ecological Station (two populations) and found greater genetic variation among populations. We suspected that the inconsistency between these studies and ours might be related to the numbers of populations and geographic location because population genetic structure arose from the interactions of the unique population‐ and species‐level processes (Wang et al., 1999). Our sampling, which included a larger number of populations and a greater portion of the geographic range of *P. villosa*, might have yielded results that are better able to detect patterns of differentiation among populations.

In our study, we used a combination of approaches including UPGMA, SAMOVA, PCoA and gap static analysis, STRUCTURE, and SplitsTree to identify the main clusters among the 43 populations of *P. villosa*, and, taken together, the results support to main clusters, Groups 1 and 2, with possible additional structuring within these. Notably, populations with closer geographic distances did not always cluster together, in the UPGMA tree, STRUCTURE, or SplitsTree. Moreover, SplitsTree, along with the PCoA, showed that some individuals from the same population did not always group together, especially individuals from populations 36, 37, 38, 39, and 43. Taken together, this may suggest that gene flow, while is rare in *P. villosa*, often occurs over long distances rather than between adjacent populations. Thus, there might be some critical, yet‐unknown dispersal vector for *P. villosa*, such as birds. However, populations 36, 37, 38, 39, and 43 occurred in the Helan Mountains, which represented a transitional zone between desert and steppe vegetation (Takhtajan, 1986) and contained a vastly richer flora than the broader, surrounding area (Jiang et al., 2007). The transitional nature of this area may be more likely to support fixation of genes introduced from outside populations; that is, among other populations outside of the Helan Mountains, dispersal of propagules may occur commonly, but selection favors local ecotypes. Overall, with limited effective gene flow, *P. villosa* has undergone considerable genetic divergence and exhibits a high level of genetic structure.

Although long‐distance dispersals might be a one critical aspect of genetic structure in *P. villosa*, genetic distance was also significantly correlated with geographic distance based on a Mantel test. Therefore, genetic structure in *P. villosa* might primarily result from geographic isolation imposed by mountains (e.g., Yin Mountains; Helan Mountains) and large deserts in northwestern China (e.g., Tengger Desert; Mu Us Sandy Land) as well as range contraction and population fragmentation induced by climatic oscillations as also observed for *Gymnocarpos przewalskii* Maxim. and *Helianthemum songaricum* Schrenk (Ma et al., 2012; Meng et al., 2014; Su et al., 2011). In addition, founder effects and population bottlenecks might have also contributed to the genetic structures of the species (Birky et al., 1989; Liu et al., 2015).

### 4.3 Demographic history of *P. villosa*

The genetic diversity within Group 2 was slightly lower than that of Group 1, despite that Group 1 comprises a smaller number of populations (12 vs. 31). Based on this, extant populations of this species might originate from the genetic stock of Group 1, as geographic areas with both high genetic diversity and frequency of dominant genes usually represent centers of origins for source populations (Vavilov, 1926). However, our study design and results cannot discern the exact center of origin for the species nor the main migrational patterns of *P. villosa*, and accomplishing this will require additional molecular data and informatics approaches.

Climate oscillation during the Quaternary has often been hypothesized to be an important factor in influencing the current geographic distribution and demographic history of plant species (Hewitt, 2004; Su & Zhang, 2013). One widely utilized approach to comparing past and future distributions of plant species and determining the primary environmental factors driving them is ENM (e.g., Bai et al., 2017; Nabout et al., 2016; Wei et al., 2018). Specifically, our models showed that the range of *P. villosa* was the most extensive during the LIG period and included the northeast edge of the Qinghai‐Tibet Plateau, Tarim Basin, Tianshan Mountains, Inner Mongolia Plateau, and the western regions of DaXinggan Ling. The range became limited to the Inner Mongolia Plateau, Ordos Plateau, and the Yinshan‐Helanshan area during the LGM. The contraction of the range is likely the result of glaciation and climatic shifts within the Tianshan Mountains and Tarim Basin, where temperatures dropped significantly as glaciation developed on a large scale in the Northern Hemisphere during the early‐Middle Pleistocene (Meng et al., 2014; Xu et al., 2010; Yi et al., 2004). Nevertheless, it is surprising that the species range did not rebound as temperatures grew warmer following the LGM. This may be because of the onset of extreme aridity within the region during the Quaternary period, as this is widely known to have played a significant role in determining the geographic distribution and evolutionary history of many plant species (Meng & Zhang, 2011; Su & Zhang, 2013; Su et al., 2011). For example, in a previous study of *Helianthemum songaricum* (Cistaceae), which occured in Northern China and adjacent desert areas of central Asia (Yang & Gilbert, 2007), the worsening of the dry climate constrained the distributional range, and acceptable habitats for the species gradually became reduced and fragmented (Su et al., 2011). Future studies of *P. villosa* may utilize genomic data and seek to understand the evolution of genes involved in adaptation to aridity.

### 4.4 Germplasm conservation of *P. villosa*

*Psammochloa villosa* is a dominant species in its desert habitat, and sometimes it is the only herbaceous species occurring within its plant community. The species helps to maintain a fragile desert ecosystem by preventing wind erosion, the development of quicksand, and further desertification (Cai, 2016). After observing populations at 43 sampling locations during our field work, we noted that some populations of the species presently grow in severely degraded habitats. While we found that *P. villosa* is a species of least concern (LC) based on EOO (2,064,370 km²), habitat degradation may be an impending threat to the species and jeopardize its vital ecological role. Therefore, we advocate for continued ecological monitoring of this dominant, keystone desert grass.

*Psammochloa villosa* may have great potential for sustainable utilization as a forage plant for livestock. The sand whips have relatively long inflorescences with large spikes that make it suitable for forage. Moreover, its adaptations to drought may make this species a valuable source of genetic resources for molecular breeding of other crop and forage species as, presently, it is one of few forage species that can withstand the intensifying long‐term drought conditions in northwest China. Developing a sustainable use strategy for *P. villosa* will also help to ensure its continued availability as a keystone species within desert communities of the Inner Mongolian Plateau and adjacent areas.

## CONFLICT OF INTEREST

None declared.

## AUTHOR CONTRIBUTIONS

**Ting Lv:** Conceptualization (equal); data curation (equal); formal analysis (equal); investigation (equal); methodology (lead); writing‐original draft (lead); writing‐review & editing (equal). **AJ Harris:** Conceptualization (equal); writing‐original draft (supporting); writing‐review & editing (equal). **Tao Liu:** Formal analysis (equal); investigation (equal); methodology (supporting). **Ruifang Liang:** Data curation (equal); formal analysis (equal); methodology (supporting). **Zilan Ma:** Formal analysis (equal); investigation (equal); methodology (supporting). **Yuping Liu:** Conceptualization (equal); formal analysis (equal); funding acquisition (lead); methodology (supporting); writing‐review & editing (equal). **Xu Su:** Conceptualization (equal); formal analysis (equal); investigation (equal); project administration (supporting); supervision (lead); writing‐original draft (supporting); writing‐review & editing (equal).

## Supporting information

Fig S1

Fig S2

Table S1‐S5

Fig S1‐S2‐captions

Supplementary File 1

## Data Availability

All tables and figures supporting the results and conclusions were included in the article, except for the binary scoring of AFLP bands, which we have submitted to the Dryad Digital Repository at https://doi.org/10.5061/dryad.dbrv15f0v.
