# Supplementary figures and images for: Population genetic structure and evolutionary history of Psammochloa villosa (Trin.) Bor (Poaceae) revealed by AFLP marker
Source: Ecol Evol. 2021 Jul 13;11(15):10258–76. doi: 10.1002/ece3.7831 (PMC8328423; doi:10.1002/ece3.7831)

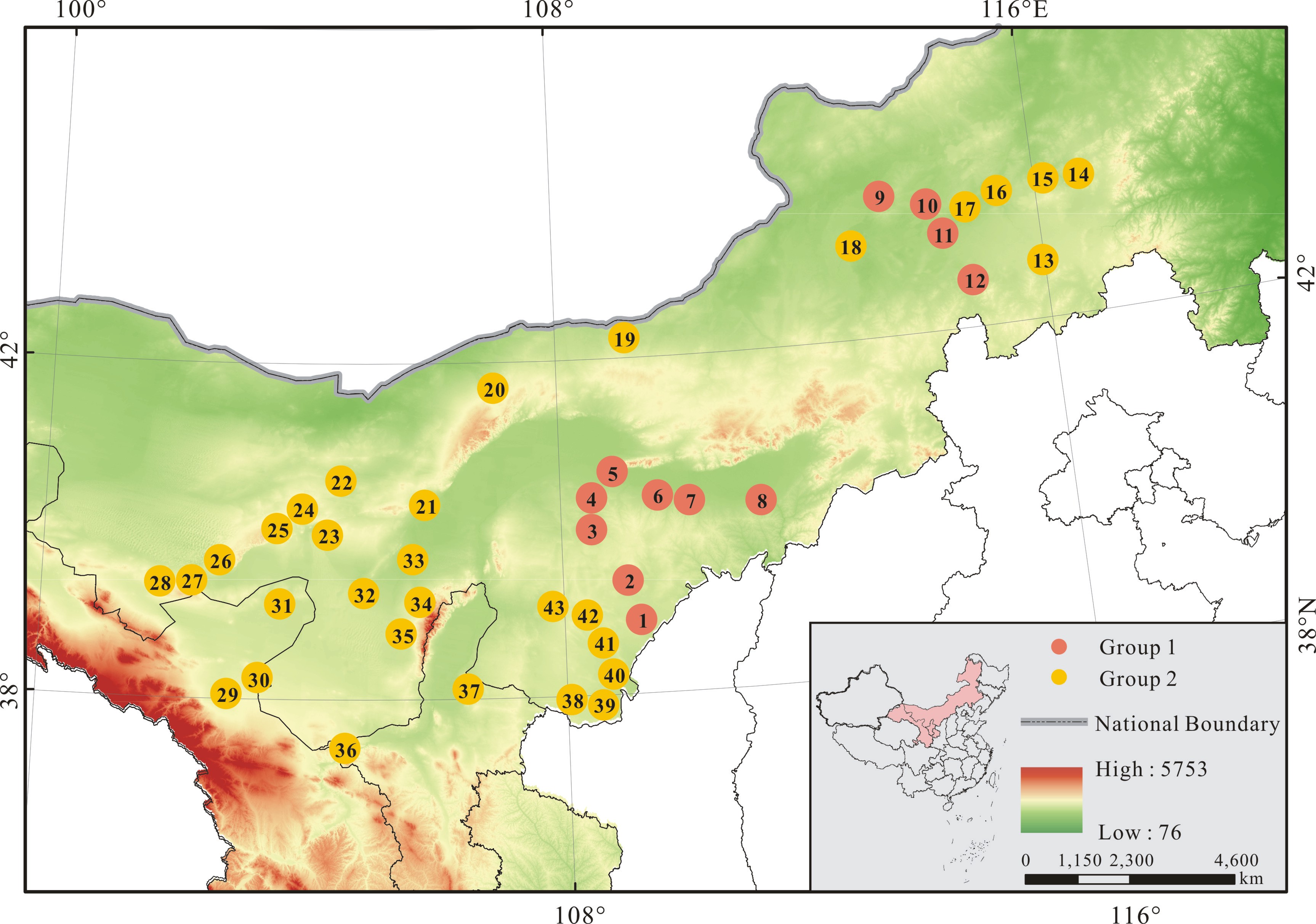

Supplement: Supplementary file 1 — Fig S1 [file ECE3-11-10258-s005.jpg]

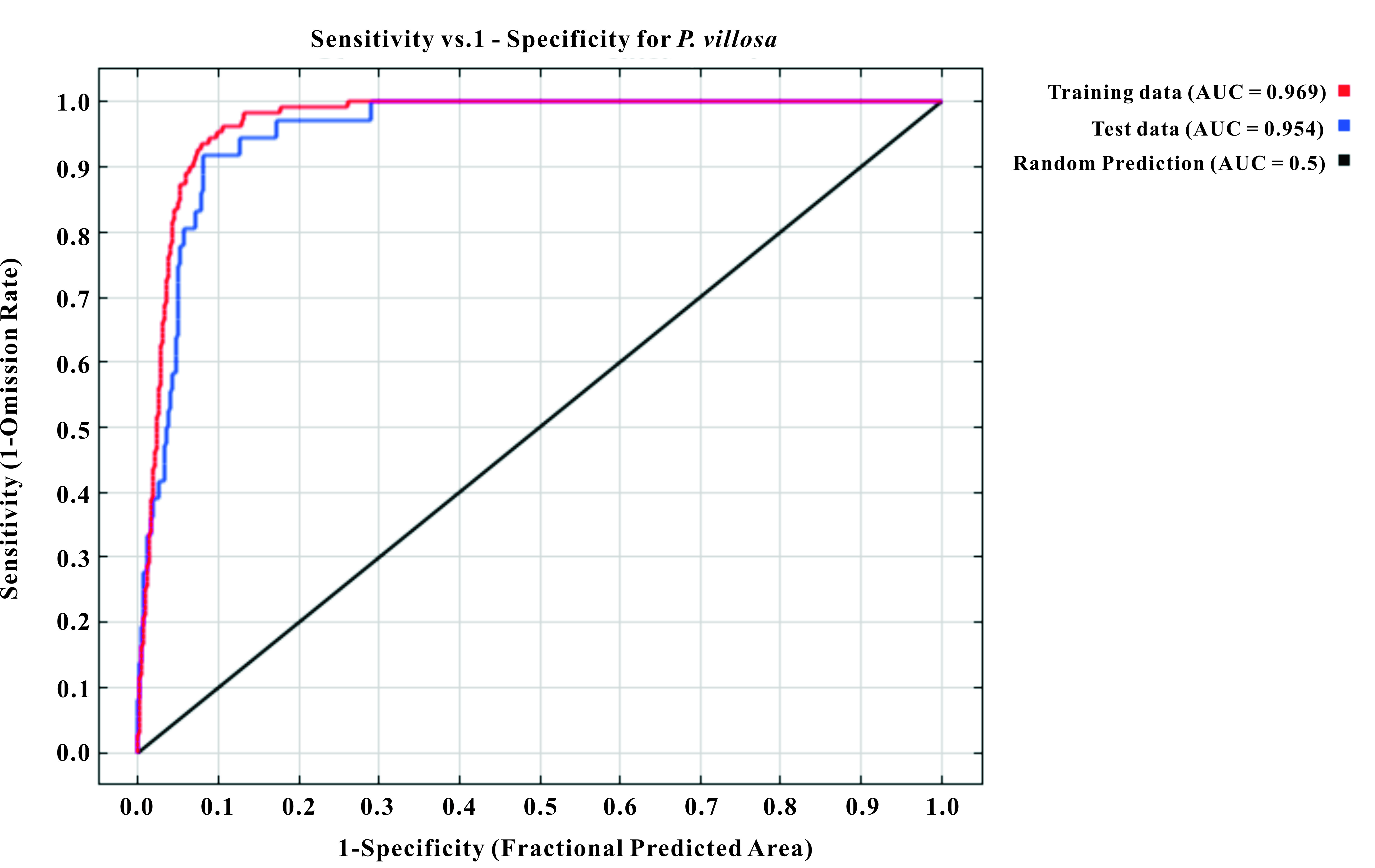

Supplement: Supplementary file 2 — Fig S2 [file ECE3-11-10258-s001.jpg]
