# Supplementary material for: Population genetic structure and evolutionary history of Psammochloa villosa (Trin.) Bor (Poaceae) revealed by AFLP marker
Source: Ecol Evol. 2021 Jul 13;11(15):10258–76. doi: 10.1002/ece3.7831 (PMC8328423; doi:10.1002/ece3.7831)
Supplement: Supplementary file 3 — Table S1‐S5 [file ECE3-11-10258-s003.docx]

**Table S1** Localities and genetic diversity indices for samples of *P. villosa* collected in the present study

| Population code | Latitude (N) | Longitude (E) | Altitude (m) | N | Polymorphic loci (%) | *N*a | *N*e | *I* | *h* | *H*e |
| --- | --- | --- | --- | --- | --- | --- | --- | --- | --- | --- |
| Populations of Group1 | | | | | | | | | | |
| 1 | 38°52′18.9" | 109°10′18.2" | 1301 | 5 | 47.1 | 0.71 | 1.18 | 0.16 | 0.12 | 0.16 |
| 2 | 39°20′37.8" | 109°00′5.9" | 1314 | 5 | 42.0 | 0.70 | 1.18 | 0.16 | 0.12 | 0.15 |
| 3 | 40°01′59.2" | 108°28′34.6" | 1236 | 5 | 42.7 | 0.65 | 1.17 | 0.15 | 0.11 | 0.15 |
| 4 | 40°12′19.5" | 108°29′11.4" | 1229 | 5 | 39.7 | 0.71 | 1.18 | 0.16 | 0.12 | 0.16 |
| 5 | 40°26'4.6'' | 108°37'52.9'' | 1114 | 5 | 35.1 | 0.64 | 1.17 | 0.15 | 0.11 | 0.15 |
| 6 | 40°20′25.8" | 109°31′21.5" | 1057 | 5 | 35.3 | 0.63 | 1.15 | 0.13 | 0.10 | 0.14 |
| 7 | 40°15′39.1" | 110°00′43.1" | 1126 | 5 | 37.6 | 0.73 | 1.18 | 0.17 | 0.13 | 0.16 |
| 8 | 40°11'57.4'' | 111°07'21.4'' | 1047 | 5 | 36.5 | 0.78 | 1.19 | 0.18 | 0.14 | 0.16 |
| 9 | 43°40′36.5" | 113°26′57.9" | 1010 | 5 | 39.4 | 0.77 | 1.20 | 0.18 | 0.14 | 0.17 |
| 10 | 43°26′4.1" | 114°18′57.8" | 1112 | 5 | 37.2 | 0.71 | 1.18 | 0.16 | 0.13 | 0.16 |
| 11 | 43°16′49.7" | 114°26′13.1" | 1035 | 5 | 39.1 | 0.78 | 1.21 | 0.19 | 0.14 | 0.17 |
| 12 | 42°33′20.9" | 114°49′0.7" | 1211 | 5 | 41.7 | 0.83 | 1.20 | 0.19 | 0.15 | 0.17 |
| Populations of Group2 | | | | | | | | | | |
| 13 | 42°41′0.0" | 115°59′0.0" | 1321 | 5 | 37.9 | 0.68 | 1.17 | 0.15 | 0.11 | 0.16 |
| 14 | 43°38′0.0" | 116°39′0.0" | 1183 | 5 | 39.2 | 0.73 | 1.19 | 0.17 | 0.13 | 0.17 |
| 15 | 43°39′0.0" | 116°10′0.0" | 1111 | 5 | 36.3 | 0.64 | 1.16 | 0.14 | 0.10 | 0.15 |
| 16 | 43°23′0.0" | 115°00′0.0" | 1056 | 5 | 41.7 | 0.78 | 1.20 | 0.18 | 0.13 | 0.18 |

**Table S1** (Continued)

| 17 | 43°18′0.0" | 114°46′0.0" | 1079 | 5 | 39.8 | 0.73 | 1.18 | 0.16 | 0.13 | 0.17 |
| --- | --- | --- | --- | --- | --- | --- | --- | --- | --- | --- |
| 18 | 43°07′11.8" | 112°54′23.5" | 1072 | 5 | 37.6 | 0.69 | 1.16 | 0.15 | 0.11 | 0.15 |
| 19 | 42°42′0.0" | 108°56′0.0" | 1323 | 5 | 37.6 | 0.67 | 1.16 | 0.14 | 0.11 | 0.15 |
| 20 | 41°41′38.0" | 107°00′7.9" | 1111 | 3 | 30.2 | 0.60 | 1.14 | 0.13 | 0.09 | 0.14 |
| 21 | 40°18′39.0" | 105°53′35.9" | 1211 | 2 | 25.7 | 0.53 | 1.13 | 0.12 | 0.10 | 0.13 |
| 22 | 40°37′0.0" | 104°35′0.0" | 1276 | 5 | 42.8 | 0.81 | 1.20 | 0.19 | 0.14 | 0.18 |
| 23 | 39°59′0.0" | 104°12′0.0" | 1243 | 5 | 40.9 | 0.75 | 1.19 | 0.17 | 0.13 | 0.17 |
| 24 | 40°07'49.5'' | 103°58'20.7'' | 1430 | 5 | 37.9 | 0.72 | 1.17 | 0.16 | 0.13 | 0.15 |
| 25 | 40°01′0.0" | 103°53′0.0" | 1403 | 5 | 44.0 | 0.83 | 1.21 | 0.19 | 0.14 | 0.18 |
| 26 | 39°24′26.3" | 102°22′1.8" | 1457 | 5 | 36.9 | 0.69 | 1.18 | 0.16 | 0.12 | 0.15 |
| 27 | 39°22'11.8'' | 102°12'52.1'' | 1570 | 5 | 35.5 | 0.66 | 1.18 | 0.15 | 0.11 | 0.15 |
| 28 | 39°21'24.6'' | 102°06'58.9'' | 1560 | 5 | 39.0 | 0.71 | 1.17 | 0.15 | 0.11 | 0.16 |
| 29 | 38°06′0.0" | 102°59′0.0" | 1498 | 5 | 39.4 | 0.72 | 1.17 | 0.16 | 0.12 | 0.16 |
| 30 | 38°13′0.0" | 103°18′0" | 1459 | 5 | 44.4 | 0.85 | 1.21 | 0.20 | 0.15 | 0.19 |
| 31 | 39°08′0.0" | 103°40′0.0" | 1311 | 5 | 42.4 | 0.78 | 1.19 | 0.17 | 0.13 | 0.18 |
| 32 | 39°16′0.0" | 104°57′0.0" | 1241 | 5 | 41.3 | 0.76 | 1.19 | 0.17 | 0.13 | 0.17 |
| 33 | 39°40′1.2" | 105°42′12.8" | 1025 | 5 | 38.9 | 0.50 | 1.14 | 0.12 | 0.10 | 0.16 |
| 34 | 38°57′43.2" | 105°39′16.3" | 1458 | 5 | 34.6 | 0.62 | 1.15 | 0.13 | 0.10 | 0.14 |
| 35 | 38°47′0.0" | 105°31′0.0" | 1179 | 5 | 42.5 | 0.79 | 1.20 | 0.18 | 0.14 | 0.18 |

**Table S1** (Continued)

| 36 | 37°25′29.1" | 104°40′5.0" | 1707 | 5 | 47.1 | 0.93 | 1.22 | 0.21 | 0.17 | 0.19 |
| --- | --- | --- | --- | --- | --- | --- | --- | --- | --- | --- |
| 37 | 38°07′9.8" | 106°30′57.8" | 1227 | 5 | 42.0 | 0.81 | 1.20 | 0.19 | 0.14 | 0.17 |
| 38 | 37°46'46.0'' | 108°08'31.4'' | 1344 | 5 | 42.7 | 0.83 | 1.20 | 0.19 | 0.14 | 0.17 |
| 39 | 37°40′52.2" | 108°50′23.5" | 1328 | 5 | 39.7 | 0.77 | 1.20 | 0.18 | 0.13 | 0.17 |
| 40 | 37°47′56.7" | 108°42′39.3" | 1254 | 5 | 35.1 | 0.63 | 1.14 | 0.13 | 0.10 | 0.14 |
| 41 | 38°22'8.1'' | 108°40'6.3'' | 1286 | 5 | 35.3 | 0.63 | 1.14 | 0.13 | 0.10 | 0.14 |
| 42 | 38°53′0.0" | 108°18′0.0" | 1371 | 5 | 40.7 | 0.75 | 1.19 | 0.17 | 0.13 | 0.17 |
| 43 | 39°01′0.0" | 108°00′0.0" | 1358 | 5 | 54.8 | 1.12 | 1.29 | 0.27 | 0.22 | 0.22 |

*N*, number of individuals; Polymorphic loci, the percentage of loci that are polymorphic out of total 1654 loci; *N*a, observed number of alleles; *N*e, effective number of alleles; *I*, Shannon's information index; *h*, Nei’s genetic diversity; *H*e, expected heterozygosity.

NX, Ningxia; NMG, Neimenggu; SX, Shaanxi. GS, Gansu.

**Table S2** Localities of 155 records used for niche modeling

| Population code | Latitude (N) | Longitude (E) | Population code | Latitude (N) | Longitude (E) | Population code | Latitude (N) | Longitude (E) |
| --- | --- | --- | --- | --- | --- | --- | --- | --- |
| P1 | 49.2126 | 119.7292 | P23 | 43.3833 | 115.0000 | P45 | 42.6736 | 115.9457 |
| P2 | 45.5126 | 116.9779 | P24 | 43.3500 | 114.8500 | P46 | 42.6363 | 114.7415 |
| P3 | 44.0894 | 113.9219 | P25 | 43.3000 | 114.7667 | P47 | 42.6167 | 115.8167 |
| P4 | 43.9390 | 116.0704 | P26 | 43.2973 | 113.0510 | P48 | 42.5558 | 114.8169 |
| P5 | 43.8576 | 113.6554 | P27 | 43.2909 | 116.6604 | P49 | 42.5167 | 115.5833 |
| P6 | 43.7481 | 113.7815 | P28 | 43.2805 | 114.4370 | P50 | 42.3414 | 115.9130 |
| P7 | 43.7000 | 117.0000 | P29 | 43.2500 | 113.0000 | P51 | 42.3375 | 115.0207 |
| P8 | 43.6833 | 116.3167 | P30 | 43.2122 | 112.9788 | P52 | 42.3289 | 115.0315 |
| P9 | 43.6768 | 113.4494 | P31 | 43.1523 | 114.4681 | P53 | 40.6167 | 104.5833 |
| P10 | 43.6749 | 113.9785 | P32 | 43.1199 | 112.9065 | P54 | 40.4358 | 106.4846 |
| P11 | 43.6667 | 113.4500 | P33 | 43.0613 | 114.5072 | P55 | 42.2390 | 115.9972 |
| P12 | 43.6500 | 116.1667 | P34 | 43.0119 | 115.7574 | P56 | 42.2153 | 111.8059 |
| P13 | 43.6460 | 111.9699 | P35 | 42.9256 | 114.5646 | P57 | 42.2025 | 116.5047 |
| P14 | 43.6333 | 116.6500 | P36 | 42.8632 | 89.1928 | P58 | 41.9912 | 116.2064 |
| P15 | 43.6281 | 113.3760 | P37 | 42.8345 | 114.5676 | P59 | 41.9569 | 101.0528 |
| P16 | 43.6167 | 115.3333 | P38 | 42.7698 | 114.6434 | P60 | 41.6939 | 107.0022 |
| P17 | 43.6000 | 115.6500 | P39 | 42.7329 | 112.6404 | P61 | 41.0834 | 107.0604 |

**Table S2** (Continued)

| P18 | 43.5833 | 115.7500 | P40 | 42.7293 | 112.6501 | P62 | 40.8836 | 107.1434 |
| --- | --- | --- | --- | --- | --- | --- | --- | --- |
| P19 | 43.5491 | 113.2569 | P41 | 42.7167 | 116.0833 | P63 | 40.7791 | 107.4050 |
| P20 | 43.5154 | 114.1718 | P42 | 42.7028 | 114.7319 | P64 | 40.7619 | 107.4222 |
| P21 | 43.4667 | 115.0833 | P43 | 42.7000 | 108.9333 | P65 | 40.7458 | 104.5031 |
| P22 | 43.4345 | 114.3161 | P44 | 42.6833 | 115.9833 | P66 | 40.7418 | 107.3821 |
| P67 | 40.3882 | 110.0001 | P92 | 39.8000 | 108.7000 | P117 | 38.8535 | 105.7036 |
| P68 | 40.3833 | 104.7167 | P93 | 39.6670 | 105.7036 | P118 | 38.8380 | 105.6981 |
| P69 | 40.3817 | 109.3359 | P94 | 39.6630 | 108.7794 | P119 | 38.8367 | 99.6144 |
| P70 | 40.3405 | 109.5226 | P95 | 39.6281 | 103.0403 | P120 | 38.8330 | 105.6476 |
| P71 | 40.3366 | 106.9958 | P96 | 39.4333 | 102.7500 | P121 | 38.7833 | 105.5167 |
| P72 | 40.3256 | 107.0045 | P97 | 39.4073 | 102.3672 | P122 | 38.7566 | 110.1787 |
| P73 | 40.3252 | 107.0029 | P98 | 39.3771 | 99.8179 | P123 | 38.7392 | 109.1013 |
| P74 | 40.3108 | 105.8933 | P99 | 39.3616 | 110.1672 | P124 | 38.6650 | 105.8017 |
| P75 | 40.3055 | 109.9368 | P100 | 39.3476 | 102.0119 | P125 | 38.6500 | 108.9333 |
| P76 | 40.2884 | 109.9427 | P101 | 39.3438 | 109.0016 | P126 | 38.6410 | 108.9262 |
| P77 | 40.2609 | 110.0120 | P102 | 39.3333 | 104.9000 | P127 | 38.6269 | 106.5652 |
| P78 | 40.2431 | 109.9507 | P103 | 39.2667 | 104.9500 | P128 | 38.6106 | 108.8343 |
| P79 | 40.2379 | 105.9169 | P104 | 39.2167 | 103.7000 | P129 | 38.6000 | 108.7667 |

**Table S2** (Continued)

| P80 | 40.2054 | 108.4865 | P105 | 39.2078 | 101.6601 | P130 | 38.5304 | 105.6553 |
| --- | --- | --- | --- | --- | --- | --- | --- | --- |
| P81 | 40.1975 | 110.7404 | P106 | 39.1333 | 103.6667 | P131 | 38.3667 | 103.2833 |
| P82 | 40.1333 | 110.5000 | P107 | 39.1129 | 109.0366 | P132 | 38.3282 | 109.7580 |
| P83 | 40.1167 | 104.0500 | P108 | 39.0962 | 108.0956 | P133 | 38.3000 | 109.7000 |
| P84 | 40.0943 | 109.0196 | P109 | 39.0167 | 108.0000 | P134 | 38.2167 | 103.3000 |
| P85 | 40.0833 | 103.9500 | P110 | 38.9620 | 105.6545 | P135 | 38.1799 | 109.0588 |
| P86 | 40.0634 | 103.9141 | P111 | 38.9463 | 107.8719 | P136 | 38.1194 | 106.5161 |
| P87 | 40.0331 | 108.4763 | P112 | 38.9333 | 108.1333 | P137 | 38.1167 | 103.1667 |
| P88 | 40.0167 | 103.8833 | P113 | 38.9167 | 105.5167 | P138 | 38.1000 | 102.9833 |
| P89 | 39.9833 | 104.2000 | P114 | 38.8833 | 108.3000 | P139 | 38.0333 | 102.8667 |
| P90 | 39.8203 | 109.9558 | P115 | 38.8719 | 109.1717 | P140 | 38.0306 | 104.8136 |
| P91 | 39.8141 | 109.9729 | P116 | 38.8642 | 105.7319 | P141 | 37.9761 | 106.3286 |
| P142 | 37.9567 | 108.7709 | P147 | 37.6812 | 108.8399 | P152 | 37.4248 | 104.6681 |
| P143 | 37.9318 | 102.6068 | P148 | 37.6789 | 108.3090 | P153 | 37.3636 | 102.8363 |
| P144 | 37.9212 | 107.9943 | P149 | 37.5000 | 102.9000 | P154 | 36.3000 | 98.1000 |
| P145 | 37.7991 | 108.7109 | P150 | 37.4445 | 104.9394 | P155 | 35.9944 | 97.8869 |
| P146 | 37.7769 | 107.3913 | P151 | 37.4426 | 104.9383 |  |  |  |

**Table S3** *F*_CT_ values for different numbers of population groups (*K*) inferred by SAMOVA algorithm based on the AFLP dataset

| *K* | Population grouping | *F*_CT_ | *P*_value |
| --- | --- | --- | --- |
| *K*=2 | (1, 2, 3, 4, 5, 6,7, 8, 9, 10, 11, 12) (13, 14, 15, 16, 17, 18, 19, 20, 21, 22, 23, 24, 25, 26, 27, 28, 29, 30, 31, 32, 33, 34, 35, 36, 37, 38, 39, 40, 41, 42, 43) | 0.215 | 0.000 |
| *K*=3 | (1, 2, 3, 4, 5, 6) (7, 8, 9, 10, 11, 12) (13, 14, 15, 16, 17, 18, 19, 20, 21, 22, 23, 24, 25, 26, 27, 28, 29, 30, 31, 32, 33, 34, 35, 36, 37, 38, 39, 40, 41, 42, 43) | 0.234 | 0.000 |
| *K*=4 | (1, 2, 3, 4, 5, 6) (7, 8) (9, 10, 11, 12) (13, 14, 15, 16, 17, 18, 19, 20, 21, 22, 23, 24, 25, 26, 27, 28, 29, 30, 31, 32, 33, 34, 35, 36, 37, 38, 39, 40, 41, 42, 43) | 0.231 | 0.000 |
| *K*=5 | (1, 2, 3, 4, 5, 6) (7, 8) (9, 10, 11, 12) (13, 14, 16, 17, 18, 19, 20, 21, 22, 23, 24, 25, 26, 27, 28, 29, 30, 31, 32, 33, 34, 35, 36, 37, 38, 39, 40, 41, 42, 43) (15) | 0.228 | 0.000 |
| *K*=6 | (1, 2, 3, 4, 5, 6 ) (7, 8, 9, 10, 11, 12) (13) (14, 15, 16, 17) (18, 19, 20, 21, 22, 23, 24, 25, 26, 27, 28, 29, 30, 31, 32, 33, 34, 35, 36, 37, 38, 39, 40, 41, 42, 43) | 0.225 | 0.000 |
| *K*=7 | (1, 2, 3, 4, 5, 6) (7, 8, 9, 10, 11, 12) (13) (14, 15, 16, 17) (18, 20, 21, 22, 24, 25, 26, 27, 28, 29, 30, 31, 33, 34, 36, 37, 38, 39, 40, 41) (19) (23, 32, 35, 42, 43) | 0.228 | 0.000 |
| *K*=8 | (1, 2, 3, 4, 5, 6) (7, 8, 9, 10, 11, 12) (13) (14, 15, 16, 17, 18, 19, 22 ) (20, 21, 24, 26, 33, 34) (23, 32, 35, 42, 43) (25, 27, 28, 29, 30, 31) (36, 37, 38, 39, 40, 41) | 0.230 | 0.000 |
| *K*=9 | (1, 2, 3, 4, 5, 6) (7, 8, 9, 10, 11, 12) (13) (14, 15, 16, 17) (23, 32, 35) (18, 20,21, 22, 24, 25, 26, 27, 28, 29, 30, 31, 33, 34 ) (19) (36, 37, 38, 39, 40, 41) (42, 43) | 0.232 | 0.000 |
| *K*=10 | (1, 2, 3, 4, 5, 6) (7, 8, 9, 10, 11, 12) (13) (14, 15, 16, 17) (18, 20, 21, 24, 26, 33) (19, 22) (23, 32, 35, 42, 43) (25, 27, 28, 29, 30, 31) (34) (36, 37, 38, 39, 40, 41) | 0.236 | 0.000 |

**Table S4 DIC statistics for three models applied to AFLP marker data set in *P. villosa***

| Grouping | Models | Dbar | Dhat | pD | DIC |
| --- | --- | --- | --- | --- | --- |
| All populations | Full | 93133.2 | 73546.9 | 19586.3 | 112719.0 |
|  | ƒ = 0 | 93521.7 | 72562.8 | 20958.9 | 114481.0 |
|  | *θ*^B^ = 0 | 183859.0 | 182316.0 | 1542.7 | 185401.0 |
| Group 1 | Full | 25225.8 | 20595.3 | 4630.5 | 29856.3 |
|  | ƒ = 0 | 25319.5 | 20480.2 | 4839.3 | 30158.7 |
|  | *θ*^B^ = 0 | 37715.8 | 36630.4 | 1085.4 | 38801.2 |
| Group 2 | Full | 66826.0 | 53502.1 | 13323.8 | 80149.8 |
|  | ƒ = 0 | 67165.4 | 53002.6 | 14162.8 | 81328.2 |
|  | *θ*^B^ = 0 | 118827.0 | 117364.0 | 1462.5 | 120289.0 |

**Table S5** Environmental variables used for modeling and percent contribution of *P. villosa*

| Code | Environment variable | Contribution/% |
| --- | --- | --- |
| Alt | Altitude | 40 |
| Bio 7 | Temperature annual range (Bio5-Bio6) | 17.2 |
| Bio 18 | Precipitation of warmest quarter | 16.7 |
| Slop | Slope | 8.3 |
| Bio 19 | Precipitation of coldest quarter | 7.2 |
| Bio 15 | Precipitation seasonality (coefficient of variation) | 4.2 |
| Bio 6 | Min temperature of coldest month | 2.4 |
| Asp | Aspect | 1.7 |
| Bio 5 | Max temperature of warmest month | 1 |
| Bio 2 | Mean diurnal range (mean of monthly (max temp-min temp)) | 0.9 |
| Bio 3 | Isothermality (Bio 2/Bio 7) (×100) | 0.3 |
