# Supplementary material for: Population genetic structure and evolutionary history of Psammochloa villosa (Trin.) Bor (Poaceae) revealed by AFLP marker
Source: Ecol Evol. 2021 Jul 13;11(15):10258–76. doi: 10.1002/ece3.7831 (PMC8328423; doi:10.1002/ece3.7831)
Supplement: Supplementary file 4 — Fig S1‐S2‐captions [file ECE3-11-10258-s002.docx]

**Figure S1** Localities of Group 1 (red), Group 2 (yellow) sampled of *P. villosa* in the present study

**Figure S2** The mean AUC of the test samples of *P. villosa* based on the MaxEnt model
