# Supplementary material for: Population genetic structure and evolutionary history of Psammochloa villosa (Trin.) Bor (Poaceae) revealed by AFLP marker
Source: Ecol Evol. 2021 Jul 13;11(15):10258–76. doi: 10.1002/ece3.7831 (PMC8328423; doi:10.1002/ece3.7831)
Supplement: Supplementary file 5 — Supplementary File 1 [file ECE3-11-10258-s004.docx]

#Comments begin with number/has sign

#You need to start with a file/table/spreadsheet of quantitative data. For example, this could be presence/absence of alleles (0/1), geographic distances, trait values, etc.

#The data should be organized so that each row is a sample (such as a species, population, etc., and each column the data for the sample)

#Thus, row 1 might be population 1 and column one could be its latitude, or pres/abs of an allele, or whatever.

#In this case, row two would be population 2, row 3 would be population 3, and so on.

data = read.table("C:/path/yourfile.txt",header=TRUE,row.names=1,sep="\t")

#This assumes that your data file is in "C:/path" directory and the file is called "yourfile.txt". Change as needed.

#This also assumes that your data have a header row, such as SAMPLE for column 1, LATITUDE for column 2, or whatever. If you do not have a header, change to header=FALSE

#This assumes that your data have row names; that is, each row is named something like POPULATION 1, POPULATION 2, etc. or SPECIES 1, SPECIES 2, etc. If not, delete row.names=1. Additionally, if your row names are, for some reason, not in column 1 of your data, you can change to row.names=5, for example, if your row names are in column 5.

#Finally, this assumes your data are separated by tabs (i.e., a csv file). If you want to load data separated by commas, set sep to "," instead of "\t".

#Sometimes you need to scale your data. For example, if your quantitative data are in different units, do scaling. By different units, I mean that if you have, say, seed weights in grams and leaf sizes in centimeters, scale.

scaled.data = scale(data)

#Now turns your data into a distance matrix. That is, use the quantitative values to get pairwise distances.

distOfScaledData = dist(scaled.data)

#Now generates the UPGMA with hclust using the average method. In hclust, average is UPGMA. Other methods exist within hclust. Use hang = -1 to make ulttrametric for classic visualization

UPGMA = hclust(distOfScaledData, method = "average")

#Now makes a quick and ugly plot to see the result

plot(UPGMA)

#Ok, now on to boostrapping.

#Do as follows to download a boostrapping package for UPGMA. When you are asked to select a "mirror" pick anyone, but one closer to you will be faster, such as in China

#If you are asked to download and install Rtools, just follow the directions given and click when prompted to add Rtools to system path

install.packages("devtools")

library("devtools")

install_github("sgibb/bootstrap")

#Now call/load the newly installed bootstrap library

library("bootstrap")

#Next we need to make a little modification, because the person who wrote the boostrap library made a little error. Just paste all the lines below into the R interpreter.

body(bootstrap)[[4]] <- substitute(v <- mclapply(seq_len(n), function(y, origin, size, fun, nco) {

current <- clust(resample(x, size = size), fun = fun)

return(calculateMatches(origin, current, nco))

}, mc.cores = 1, origin = origin, size = ncol(x), fun = fun,

nco = ncol(origin)))

#Next some redundant things to make this function happy

createHclustObject = function(x)hclust(dist(x), "average")

UPGMA = createHclustObject(data) #Note: If you scaled your data, replace "data" here with "scaled.data"

# Bootstrap your data

b = bootstrap(data, fun=createHclustObject, n=100L) #again, change data to scaled.data if you scaled the data; also change 100 to a higher number of you want more boostrap replications

# Plot your UPGMA

plot(UPGMA, hang=-1) #hang=-1 lines the terminals up to one another as is common for UPGMA in our field.

# Add bootstrap values to nodes in the plot

bootlabels.hclust(UPGMA, b, col="blue")
